# Metformin Induces a Dietary Restriction–Like State and the Oxidative Stress Response to Extend *C. elegans* Healthspan via AMPK, LKB1, and SKN-1

**DOI:** 10.1371/journal.pone.0008758

**Published:** 2010-01-18

**Authors:** Brian Onken, Monica Driscoll

**Affiliations:** Department of Molecular Biology and Biochemistry, Rutgers University, Piscataway, New Jersey, United States of America; Brown University, United States of America

## Abstract

Metformin, a biguanide drug commonly used to treat type-2 diabetes, has been noted to extend healthspan of nondiabetic mice, but this outcome, and the molecular mechanisms that underlie it, have received relatively little experimental attention. To develop a genetic model for study of biguanide effects on healthspan, we investigated metformin impact on aging *Caenorhabditis elegans*. We found that metformin increases nematode healthspan, slowing lipofuscin accumulation, extending median lifespan, and prolonging youthful locomotory ability in a dose-dependent manner. Genetic data suggest that metformin acts through a mechanism similar to that operative in eating-impaired dietary restriction (DR) mutants, but independent of the insulin signaling pathway. Energy sensor AMPK and AMPK-activating kinase LKB1, which are activated in mammals by metformin treatment, are essential for health benefits in *C. elegans*, suggesting that metformin engages a metabolic loop conserved across phyla. We also show that the conserved oxidative stress-responsive transcription factor SKN-1/Nrf2 is essential for metformin healthspan benefits in *C. elegans*, a mechanistic requirement not previously described in mammals. *skn-1*, which functions in nematode sensory neurons to promote DR longevity benefits and in intestines for oxidative stress resistance lifespan benefits, must be expressed in both neurons and intestines for metformin-promoted healthspan extension, supporting that metformin improves healthy middle-life aging by activating both DR and antioxidant defense longevity pathways. In addition to defining molecular players operative in metformin healthspan benefits, our data suggest that metformin may be a plausible pharmacological intervention to promote healthy human aging.

## Introduction

One of the central goals of current aging research is the identification of chemical or genetic manipulations that lower the incidence of age-related disease and degeneration by promoting youthful physiology. A candidate drug for achieving this extended healthspan is metformin, one of a class of biguanides widely used to treat type-2 diabetes and linked to promoting a broad range of health benefits by lowering the risks associated with the metabolic syndrome [1]. Notably, diabetic and cardiovascular disease patients who are prescribed metformin have increased rates of survival [2].

There are some reports that metformin can extend healthspan in non-disease mammals [3]–[5], but the nature of metformin's health-promoting effects is not well understood. Several lines of evidence support that metformin may act by inducing dietary restriction (DR) metabolism, a physiological state triggered by low caloric intake that increases lifespan, delays age-associated functional decline, and limits onset of age-associated disease across species [6]–[13]. In mammals, both metformin-treated and DR animals have low blood glucose and insulin levels, and glucose uptake in muscles is increased [14]–[16]. In addition, both metformin treatment and DR promote fatty acid usage in animals, leading to lowering of fat mass [14], [16], [17]. That metformin triggers DR-like effects is further supported by the finding that gene expression patterns in metformin-treated animals are similar to those of long-term DR mice [18].

A potential mediator of metformin benefits is the AMP-activated kinase AMPK, which functions as a cellular energy sensor [19]–[21]. Metformin activates AMPK in mammals, and activated AMPK is required for the metformin-induced lowering of hepatic glucose production and increased glucose uptake in skeletal muscles [19]–[21]. Metformin also requires the protein-threonine kinase LKB1, which activates AMPK, to lower blood glucose levels [22]. Neither LKB1 nor AMPK is thought to be a direct target of metformin, and the key upstream point of interaction is not known. Overall, then, understanding of molecular mechanisms of metformin action *in vivo* remains vague. In particular, there is a paucity of data on the benefits of metformin for non-diabetics and dissection of the proposed mechanisms for metformin modulation of healthspan in genetic models is lacking.

The nematode *Caenorhabditis elegans* is a powerful model system for defining genetic and pharmacological interventions promoting longevity and healthy aging, often by mechanisms that appear conserved across phyla [13], [23], [24]. *C. elegans daf* mutants that alter the formation of the long-lived dauer state via the insulin-like signaling pathway live longer and more youthfully than wild-type [25], [26]. In this conserved signaling pathway, *daf-2* encodes an insulin/IGF-1 receptor ortholog that activates a kinase cascade that includes the AGE-1 PI3 kinase to modulate phosphorylation of the DAF-16/FOXO family transcription factor [25]–[30]. Low signaling activity in this pathway enables the non-phosphorylated DAF-16 to translocate to the nucleus to regulate expression of lifespan-promoting gene batteries [28], [31], [32]. Another conserved longevity pathway is dietary restriction - food limitation and genetic mutations that impair feeding or nutrient uptake can increase *C. elegans* lifespan [8], [13], [33]–[39]. Several methods of food limitation extend lifespan independently of DAF-16 [33], [35]–[39], and DR further extends the already long lifespan of *daf-2* mutants, suggesting that DR increases longevity in a manner that is independent of insulin signaling [33], [39]. However, induction of *C. elegans* DR by limiting the amount of bacteria on plates suggests a lifespan-extending mechanism that is DAF-16-dependent [34], indicating that distinct food deprivation regimens may signal through different pathways to impact lifespan.

Pharmacological interventions that might induce healthy DR-like metabolism are of considerable medical interest. Here we document the effects of the putative mammalian DR mimetic metformin on *C. elegans* lifespan and healthspan. We demonstrate that metformin increases median lifespan via a mechanism that is independent of the insulin-like signaling pathway but shares genetic and phenotypic features of DR. Healthspan benefits of metformin on wild type animals require the activity of the AMP-activated kinase catalytic subunit AAK-2, its upstream kinase LKB1/PAR-4 and, unexpectedly, transcription factor SKN-1/Nrf expressed both in ASI neurons and intestine. Our data support that metformin must engage both DR and oxidative stress pathways to extend healthspan. Several aspects of metformin action appear conserved across phyla, and thus we suggest the *C. elegans* model can be exploited to identify metformin-related biguanides that convey optimal healthspan benefit and anti-aging effects.

## Results

### Metformin Treatment Extends *C. elegans* Median Lifespan and Promotes Youthful Mobility Late into Adult Life

**Figure 1:**
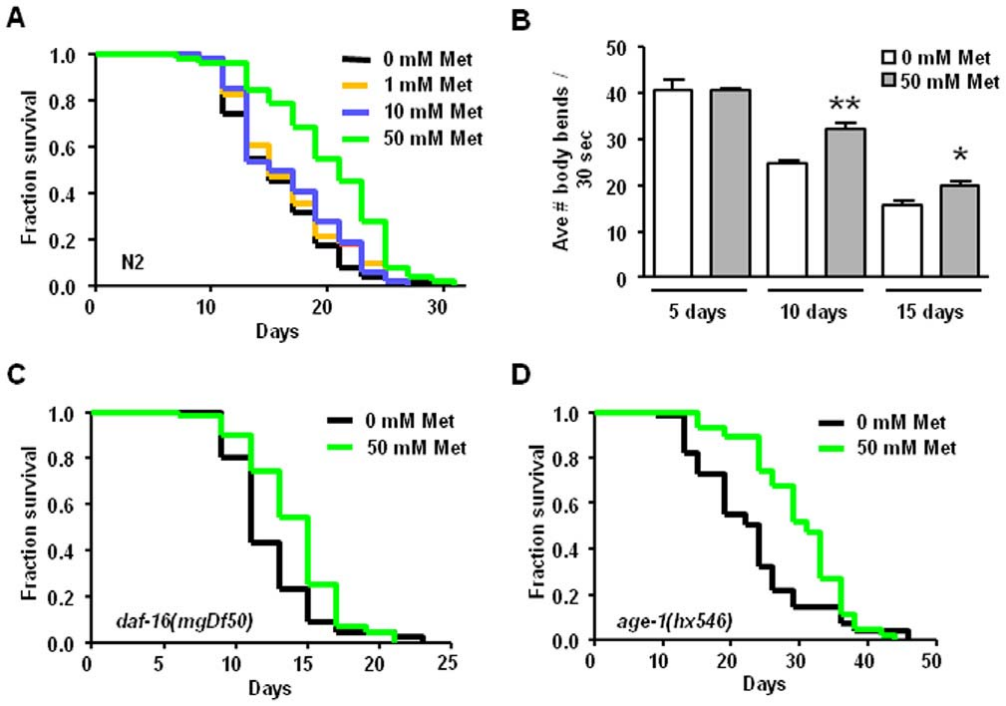
Metformin increases healthspan without a requirement for function of the insulin signaling pathway. **A.** Survival curves of wild-type (N2) animals raised at 20°C on nematode growth media plates containing either no metformin or final concentrations of 1 mM, 10 mM, or 50 mM metformin. (Note that *C. elegans* has a highly protective cuticle and intestinal lining that generally limit drug uptake such that it is not unusual for polar drugs to be applied at a concentration 1000 fold higher than their predicted affinity for the target [94], [95]; physiological levels of drug in the animals are anticipated to be much lower). For the trial presented here, median survival for animals on 0 mM, 1 mM, 10 mM, and 50 mM metformin plates was 15, 15, 17, and 21 days, respectively. The survival curves of the nematodes raised on 0 mM, 1 mM, and 10 mM metformin plates are not significantly different, while the survival curve for animals raised on 50 mM metformin is significantly different than the 0 mM metformin control (*P*<0.0001 by the Log-rank (Mantel-Cox) test; benefits are lost at 100 mM metformin, data not shown). The pooled data for all trials show an approximately 27% increase in median lifespan with 50 mM metformin treatment and a significantly right-shifted survival curve (*P*<0.0001 by the Log-rank test), see Table S1A. All studies documented here involved life-long metformin exposure, but a preliminary trial in which metformin was introduced at the last larval stage suggests that treatment in adult life is sufficient to confer some lifespan benefit (data not shown). **B.** Swimming rates for wild-type animals raised from eggs on plates (time 0) containing either no metformin or 50 mM metformin at 20°C. We recorded the number of body bends/30 seconds for individual animals placed in liquid media at early adulthood (5 days), mid-life (10 days), and late life (15 days). We present the averages of three independent trials. Metformin-treated animals swim at similar rates in young adulthood, supporting that metformin does not induce hyperactive swimming or confer developmental defects, but the swimming rates of animals raised on 50 mM metformin are significantly higher than those of the control group on days 10 and 15 (*P* = 0.0058 and *P* = 0.0346, respectively, by unpaired t tests), indicating extended mid-life and late-life locomotory ability consequent to metformin treatment. **C.** Survival curves of null mutant *daf-16(mgDf50)* raised on 0 mM and 50 mM metformin plates at 20°C. The median survival is 11 and 15 days for animals raised on 0 mM and 50 mM metformin, respectively, and the survival curves of the two groups are significantly different (*P* = 0.0111 by the Log-rank test). 1 mM and 10 mM metformin do not significantly change the survival curves of *daf-16(mgDf50)* animals (data not shown). We performed this experiment a total of four times with similar results (pooled data show a 15% median lifespan increase and a significantly right-shifted survival curve (*P*<0.0001, Log-rank) for animals treated with 50 mM metformin, see Table S1B for additional data). **D.** Survival curves of *age-1(hx546)* mutants raised on 0 mM and 50 mM metformin plates at 20°C. Median survival for animals on 0 mM and 50 mM metformin is 24 and 31 days, respectively, and the survival curves are significantly different by the Log-rank test (*P* = 0.0014). We found similar median survival extension in a single repeat of this experiment (pooled data for the two trials show an approximate 36% median lifespan increase and significantly different survival curves (*P*<0.0001, Log-rank), see Table S1C).

Despite the considerable potential for metformin and related drugs to be utilized to promote healthy aging [1]–[5], [40]–[42], the molecular mechanisms by which metformin confers healthspan benefits have not been experimentally dissected. To address this gap in understanding, we first asked whether metformin could induce physiological outcomes in *C. elegans* similar to those reported for mammals. We monitored the survival rates of *C. elegans* raised on increasing concentrations of metformin (Fig. 1A). We found that wild-type cultures raised on 1 mM and 10 mM metformin display survival curves that are similar to those of control populations raised in the absence of metformin. Wild-type animals raised on 50 mM metformin, however, show an approximately 40% increase in median survival (21 days vs. 15 days for the mock-treated control) and exhibit a significantly right-shifted survival curve (*P*<0.0001 by the Log-rank (Mantel-Cox) test; similar results were observed in 10 out of 12 trials performed with wild-type animals throughout the course of this study; Table S1A). Interestingly, the effects of metformin are most pronounced in mid-life without maximum lifespan extension, indicating that at this concentration, metformin treatment specifically impacts median lifespan of the culture.

As *C. elegans* ages, locomotion rates on solid media and in liquid decline [43]–[46], and the severity of impairment is roughly correlated with the degree of muscle deterioration that bears striking cell biological similarity to human sarcopenia [43], [47]. To ask whether metformin can also increase quality of life by extending locomotory healthspan, we tested the effects of 50 mM metformin on the decline in swimming vigor (quantitated as body bends per unit time in a liquid environment) in aging populations (Fig. 1B). We found that early in adulthood, there is no significant difference between the swimming rates of animals raised in the absence of metformin and those raised on 50 mM metformin. Thus, metformin does not induce developmental defects that impact locomotory ability, nor does it cause hyperactive swimming. In middle-aged 10 day-old animals, however, animals raised on 50 mM metformin display significantly higher swimming rates as compared to the non-treated control group. By day 15 of life, swimming rates decline further in both groups, and, although the difference in the average number of body bends between the two groups is not as great as on day 10, the metformin-treated animals are still significantly better swimmers late into adulthood. Thus, metformin extends locomotory healthspan, with the greatest impact exhibited in mid-life. Taken together, results in Fig. 1A and 1B demonstrate that metformin extends mid-life viability and slows locomotory decline, supporting that metformin promotes and/or extends youthful physiology in *C. elegans.*

### Metformin Extends Median Lifespan Independently of the Insulin Signaling Pathway

Downregulation of the insulin signaling pathway increases *C. elegans* lifespan and locomotory healthspan in a FOXO/DAF-16-dependent manner [25], [26], [44], [48]. To investigate whether metformin extends median lifespan by acting through the insulin signaling pathway, we tested a *daf-16* null mutant strain that lacks the FOXO transcription factor needed for the longevity effects of the insulin signaling pathway (Fig. 1C). We found that, like wild-type, *daf-16(mgDf50)* mutants raised on 50 mM metformin (but not on 1 or 10 mM metformin) display a median lifespan extension (11 days for the *daf*-*16* deletion mutant vs. 15 days with metformin treatment (*P* = 0.0111 by the Log-rank test), an approximately 36% increase with metformin; see Table S1B for details on these data and similar results from three other trials). We conclude that metformin can extend median lifespan via a mechanism that is independent of DAF-16/FOXO, consistent with the conclusion that metformin exerts healthspan benefits via a mechanism distinct from the insulin-like signaling pathway.

To further address requirements for insulin signaling in metformin action, we tested for metformin effects on the lifespans of insulin signaling pathway mutants. We raised long-lived *age-1(hx546)* mutants deficient in PI-3 kinase signaling in the absence or presence of metformin (Fig. 1D). We found that 50 mM metformin treatment extends median lifespan in the *age-1* mutant (30 days with metformin treatment vs. 21 days for the *age-1* control (*P* = 0.0014 by the Log-rank test), an approximate 43% increase). Similar to treated wild-type and *daf-16(mgDf50)* animals, the *age-1* median lifespan, but not the maximum lifespan, is increased by metformin treatment. We observed similar results to those depicted in Fig. 1D in a repeat trial using *age-1(hx546)* animals and the same trend in a trial using the long-lived *daf-2(e1370)* mutant strain (Table S1C). That metformin effects on lifespan are additive with low insulin signaling mutants suggests that metformin might act primarily in a pathway parallel to that of insulin signaling to confer health benefits.

### Metformin Treatment Does Not Extend Median Lifespan in the Eat-2 DR Model, Suggesting Healthspan Benefits May Be Conferred via a DR Pathway

**Figure 2:**
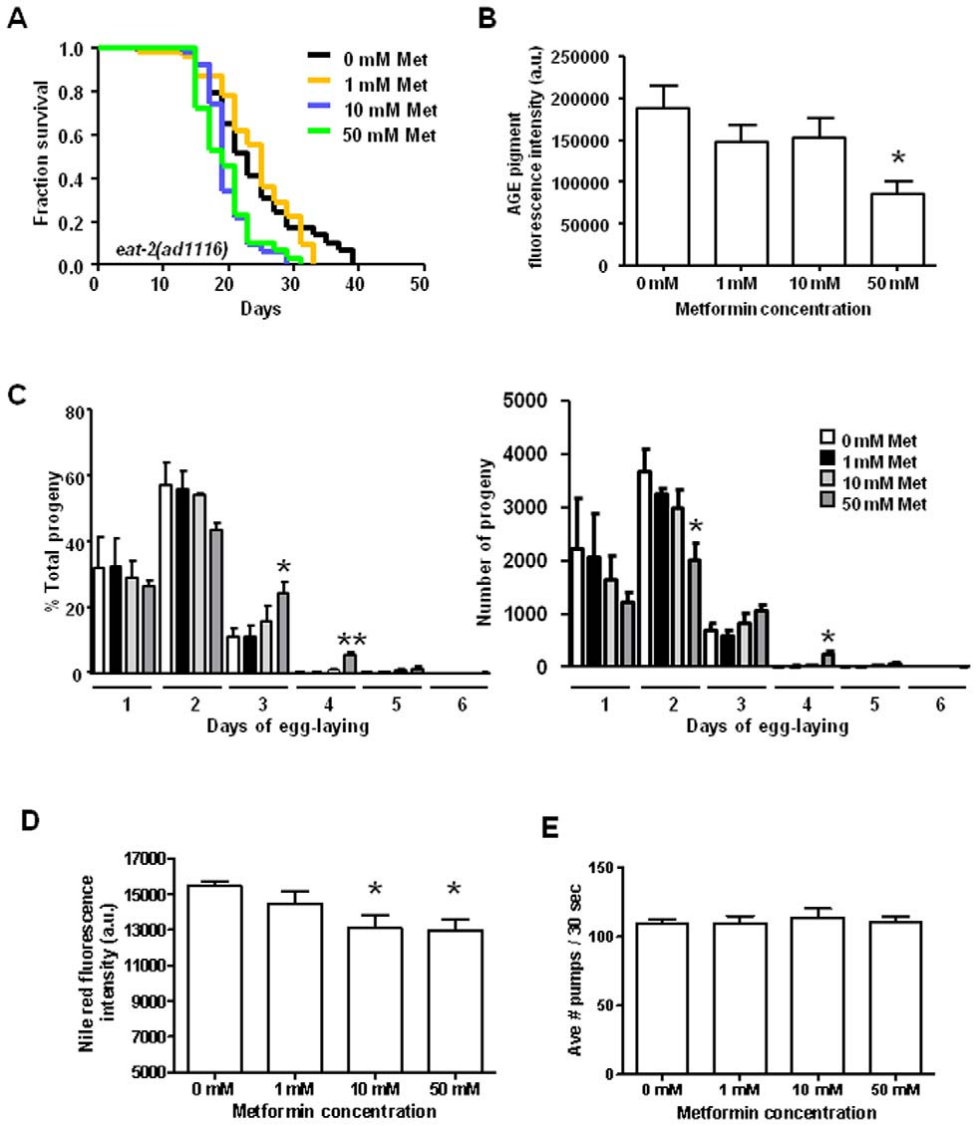
Metformin does not further increase the median lifespan of DR-constitutive mutant eat-2, and metformin treatment triggers several DR phenotypes in wild-type *C. elegans* without impairing feeding. **A.** Survival curves of *eat-2(ad1116)* mutants raised on 0 mM, 1 mM, 10 mM, and 50 mM metformin plates at 20°C. The median survival is 23, 25, 19, and 19 days for 0 mM, 1 mM, 10 mM, and 50 mM metformin, respectively. The survival curves of the animals raised on 0 mM and 1 mM metformin are not significantly different, but survival of the 10 mM and 50 mM groups are significantly reduced compared to controls (*P* = 0.0050 and 0.0033, respectively, by the Log-rank test). We performed this experiment a total of four times, with two of the trials showing significantly different survival curves for the 10 mM and 50 mM groups. Pooled data show approximately 9% declines in median lifespan for 10 mM and 50 mM metformin treatment and significant shifts in survival curves (*P = *0.0037 and 0.0114 for 10 mM and 50 mM metformin treatment, respectively, by the Log-rank test), see Table S1D. **B.** Age pigment fluorescence measurements in wild-type animals raised on 0 mM, 1 mM, 10 mM, and 50 mM metformin plates. We found that age pigment fluorescence decreases with increasing metformin concentration with the levels in the 50 mM group significantly lower than the controls (*P* = 0.0270 by an unpaired t test). Scores are the average age pigment fluorescence intensity levels of three independent trials. We also observed shifts in the excitation wavelength corresponding to peak age pigment fluorescence intensity in animals treated with 100 mM metformin (see Fig. S1). **C.** Progeny profiles of wild-type animals raised on 0 mM, 1 mM, 10 mM, and 50 mM metformin. We recorded the number of progeny produced by 60 individuals for each group for each day of egg-laying. We found no significant differences between the progeny profiles of the 0 mM, 1 mM, and 10 mM metformin groups. The 50 mM metformin-treated animals, however, exhibit delays in egg-laying, with significantly higher percentages of total progeny produced on days 3 and 4 compared to the controls (*P* = 0.0431 and 0.0084 for days 3 and 4, respectively, by an unpaired t test). The 50 mM group also shows lower levels of progeny production on days 1 and 2 compared to controls, with the levels on day 2 being significantly different (*P* = 0.0361 by an unpaired t test). Data shown represent the average of three independent trials. **D.** Quantitation of Nile Red staining of lipid deposits in wild-type animals raised on 0 mM, 1 mM, 10 mM, and 50 mM metformin. Fluorescence levels decrease with increasing metformin concentration, with significantly lower levels in the 10 mM and 50 mM metformin groups (*P* = 0.0400 and 0.0205 for 10 mM and 50 mM metformin, respectively, by an unpaired t test). Data represent the average of three independent experiments. Animals not stained with the Nile Red dye produced no detectable levels of fluorescence in this wavelength range (data not shown). **E.** Pharyngeal pumping rates of wild-type animals raised on 0 mM, 1 mM, 10 mM, and 50 mM metformin. We recorded pumping rates of 30 individuals for 30 seconds on day 4 of life, and the averages of three separate experiments are shown. Pharyngeal pumping did not differ significantly for any of the groups (*P* = 0.9231, 0.1689, and 0.9142 for 1 mM, 10 mM, and 50 mM metformin, respectively, by unpaired t tests).

Having ruled out that metformin confers major healthspan benefits via the insulin signaling pathway, we considered the possibility that metformin increases median lifespan by triggering DR-like physiology, a plausible hypothesis given suggestions that metformin acts as a DR mimetic in mammals [4], [5], [18]. We predicted that if metformin triggers a DR-like metabolic state, drug treatment would not greatly increase median lifespan of animals already undergoing DR. To address this hypothesis, we tested the effects of metformin treatment on *eat-2(ad1116)*, a long-lived strain that is defective in pharyngeal pumping and is considered DR-constitutive due to its impaired ability to feed [33] (Fig. 2A). We were unable to identify any concentration of metformin that could extend median lifespan in the *eat-2* background. Notably, in two out of four trials, doses of metformin that are effective in increasing median lifespan in other tested strains were actually detrimental to the viability of *eat-2* mutants (Fig. 2A and Table S1D). In the trial shown in Fig. 2A, metformin at 10 mM and 50 mM decreases *eat-2* median lifespan (19 days for both 10 mM and 50 mM in *eat-2* vs. 23 days for the control; *P* = 0.0050 and *P* = 0.0033, respectively, by the Log-rank test). That metformin does not further increase median lifespan in a DR-constitutive genetic background suggests that metformin acts through a DR mechanism. Additionally, because high doses of metformin can result in reduced lifespan in *eat-2* mutants, it appears that when an animal is already in the DR state, further stimulation of DR physiology by metformin leads to diminishing, even harmful, effects, a phenomenon typical of extreme calorie limitation or starvation in *C. elegans* [34]–[36], [39] and mammals [49].

### Metformin Treatment Induces DR-Associated Features in *C. elegans*

Given the genetic indication that metformin extends median lifespan via a DR mechanism, we examined metformin-treated animals for other features characteristic of DR animals. In *C. elegans*, DR is linked to a distinctive fluorescent age pigment profile, contributed by advanced glycation end products and lipofuscin, that accumulate in intestinal lysosomes [50]. Animals in the DR state have low levels of age pigment fluorescence, and they display a DR-specific shift in the excitation maximum for peak age pigment fluorescence early in life. We therefore measured age pigments in wild-type animals raised on metformin in order to see if the drug could trigger the DR-associated age pigment fluorescence profile. As shown in Fig. 2B, age pigment levels decrease with increasing levels of metformin treatment, with 50 mM metformin significantly lowering age pigment fluorescence levels. We also noted the shift in age pigment excitation maximum wavelengths at high metformin concentrations (Fig. S1). Thus, metformin treatment induces age pigment features that uniquely characterize the DR state.

Another characteristic of DR *C. elegans* is an extended period of egg-laying with an overall reduction in progeny production as compared to well-fed animals [51]. In order to examine whether metformin affects *C. elegans* reproduction, we measured egg-laying and progeny production of metformin-treated animals (Fig. 2C). We found that 50 mM metformin extends the number of days that wild-type hermaphrodites lay eggs, resulting in approximately one extra day of egg-laying. In addition, in the early period of egg-laying, the relative number of progeny produced per day is decreased in metformin-treated animals. Thus, metformin impacts reproduction in a manner typical of DR.

Feeding-defective strains undergoing DR tend to be smaller than well-fed worms and have decreased levels of fat deposits [52]. We examined overall fat levels in wild-type animals raised in the presence or absence of metformin using Nile Red, a fluorescent vital stain that has been used to measure lipid deposits [53], [54]. We quantitated Nile Red fluorescence *in vivo* using a spectrofluorimeter and methods similar to those used to measure age pigment levels (age pigments are measured at excitation wavelengths between 280–410 nm and an emission wavelength of 430 nm, while Nile Red fluorescence is measured at an emission wavelength of 658 nm and an excitation wavelength of 578 nm, so measurements of age pigments and fat can be made independently, without cross-interference). As shown in Fig. 2D, animals raised in the presence of 10 mM and 50 mM metformin exhibit lower levels of Nile Red fluorescence as compared to controls. Thus, like *C. elegans* under DR, metformin decreases levels of stored fat.

### Metformin Does Not Directly Impact Feeding Proficiency

One trivial mechanism by which metformin could trigger DR in *C. elegans* could involve lowering the pharyngeal pumping rate, which could induce DR by limiting feeding (akin to what is thought to occur in *eat-2* animals). To rule out this possibility, we measured pharyngeal pumping in wild-type animals raised in the presence of metformin (Fig. 2E). We found no significant differences in pharyngeal pumping in animals treated with metformin compared to non-treated controls, indicating that metformin is unlikely to directly limit feeding capacity.

Overall, our genetic data support that healthspan benefits of metformin are induced via DR rather than insulin signaling pathways. We find that metformin triggers multiple indicators of DR (increased median lifespan, extended locomotory healthspan, delayed egg-laying, lowered progeny production, lowered age pigment levels, and decreased fat) in a dose-dependent manner. These data support the hypothesis that metformin activates DR physiology in well-fed *C. elegans*, and, given mammalian data indicating that metformin induces features of DR [14]–[18], suggest that metformin acts via a conserved metabolic mechanism to extend healthspan.

### Metformin-Induced Extended Median Lifespan Requires AMPK

**Figure 3:**
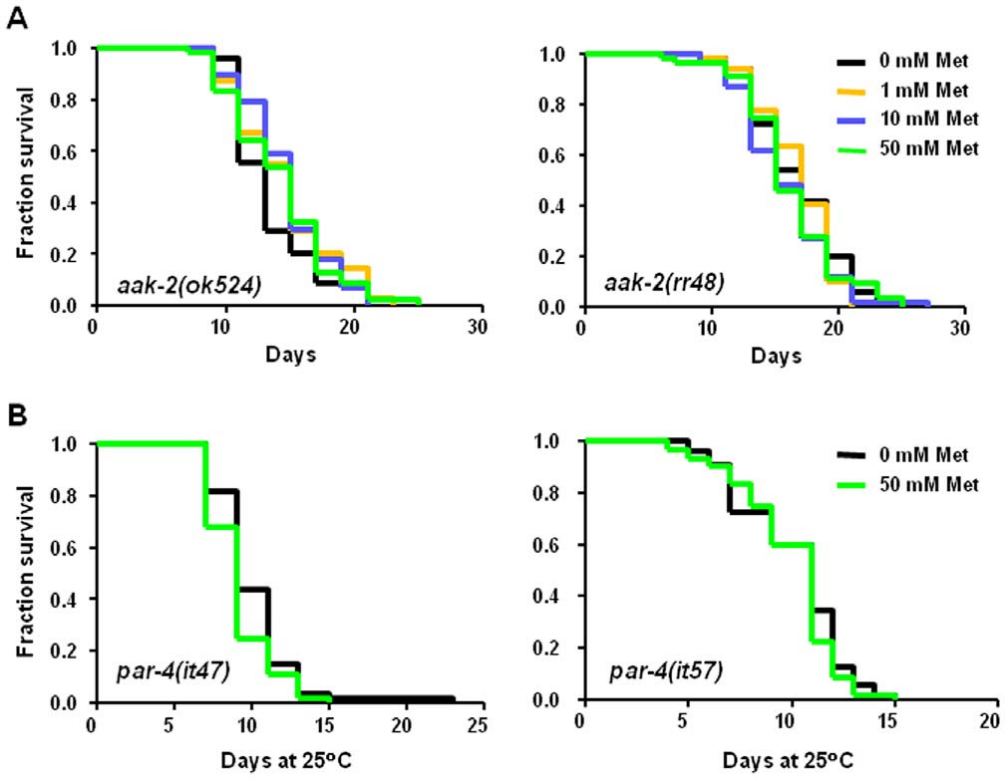
Metformin extends median lifespan via an AMPK and LKB1-dependent mechanism. **A.** Survival curves of AMPK catalytic subunit mutants *aak-2(ok524)* and *aak-2(rr48)* raised on metformin plates at 20°C for their entire lives. Median survival for *aak-2(ok524)* animals on 0 mM, 1 mM, 10 mM, and 50 mM metformin is 17, 17, 15, and 15 days, respectively, and there are no significant differences between any of the survival curves (Log-rank test). Median survival for *aak-2(rr48)* animals on 0 mM, 1 mM, 10 mM, and 50 mM is 13, 15, 15, and 15 days, respectively, and there are no significant differences between the survival curves. Pooled data from three independent trials for each *aak-2* strain show approximately 11% declines with 10 mM and 50 mM metformin treatment in *aak-2(ok524)* animals, and approximately 6% and 19% declines with 10 mM and 50 mM metformin treatment, respectively, in *aak-2(rr48)* animals. Survival curves are significantly shifted to the left in *aak-2(ok524)* animals treated with 10 mM and 50 mM metformin (*P* = 0.0209 and <0.0001, respectively), and in *aak-2(rr48)* animals raised on 10 mM and 50 mM metformin (*P* = 0.0442 and 0.0004, respectively). See Table S1E for additional data. We conclude that AMPK activity is needed for median lifespan extension induced by metformin, and that metformin has detrimental lifespan effects in the absence of AMPK. **B.** Survival curves of temperature-sensitive *lkb-1/par-4* mutants grown on 0 mM and 50 mM metformin plates, maintained at 15°C and shifted to 25°C at the L4 stage. *C. elegans par-4* encodes an ortholog of mammalian LKB1, which has been shown to activate AMPK in both mammals and nematodes. Median survival is 9 days for the *par-4(it47)* mutants grown on both 0 mM and 50 mM metformin, and there is no significant difference between the survival curves by the Log-rank test. Similarly, *par-4(it57)* animals grown on both 0 mM and 50 mM metformin have a median survival of 11 days, and their survival curves are not significantly different by the Log-rank test. Pooled data from three independent trials using the *par-4(it47)* strain show no differences in median lifespan for 0 mM vs. 50 mM metformin-treated animals (9 days for both conditions), although the 50 mM metformin survival curve is significantly shifted to the left (*P* = 0.0500, Log-rank; Table S1F). Pooled data for four independent trials with the *par-4(it57)* mutants show a decrease in median lifespan (9 days for 50 mM metformin plates vs. 10 days for 0 mM metformin; Table S1F) and a significantly left-shifted survival curve for 50 mM metformin-treated animals (*P* = 0.0063, Log-rank; Table S1F). Thus, LKB1 is required for metformin to increase median lifespan, and, as is seen in the absence of AMPK, metformin has harmful effects when LKB1 is disrupted.

We next took genetic approaches toward identifying molecules that mediate metformin healthspan benefits in physiological context. In mammals, energy sensor AMPK is activated by metformin [20], [21], and metformin-activated AMPK increases glucose uptake in muscle and inhibits gluconeogenesis in hepatocytes [19]. *C. elegans aak-2* encodes one of two homologs of the catalytic α subunit of the AMPK heterotrimeric complex, and nematode AAK-2 is activated by AMP, as occurs for mammalian AMPK [55]. Overexpression of the catalytic AMPK α subunit AAK-2 increases *C. elegans* longevity [55], and *aak-2* is required for the increased lifespan of several longevity mutants [56] and for lifespan extension under at least one tested mode of DR induction [34]. To investigate the role of AMPK in metformin-induced extended median lifespan in *C. elegans*, we tested for metformin effects in the backgrounds of two independent *aak-2* alleles: *aak-2(ok524)*, a presumed molecular null [55], and *aak-2(rr48)*, which carries a point mutation predicted to disrupt the catalytic activity of the α subunit [57]. As shown in Fig. 3A, no tested metformin concentration significantly increased the median lifespan of either *aak-2* mutant (in three repeated trials for each strain; Table S1E). Instead, metformin treatment decreased mid-life viability in *aak-2* mutants in a dose-dependent manner (Table S1E) and reduced locomotory ability in both *aak-2* strains (Fig. S2A). We conclude that the catalytic AMPK subunit is required for median lifespan extension and locomotory healthspan increase in metformin-treated *C. elegans*. Moreover, we note that in the absence of healthspan-promoting AMPK activity, metformin confers deleterious consequences on culture survival and swimming prowess. Interestingly, although we did not note statistically significant differences in Nile Red staining (Fig. S2B), we did find lower age pigments in *aak-2(ok524)* mutants treated with 50 mM metformin vs. controls (Fig. S2C, other allele not tested). This observation suggests age pigment changes might be conferred via an AMPK-independent mechanism.

### Kinase LKB1 Is Required for Metformin Healthspan Benefits

AMP is known to activate AMPK via two mechanisms. First, AMP binds allosterically to activate AMPK ∼5-fold [58]. Second, and likely more importantly, AMP binding promotes AMPK α subunit phosphorylation by the LKB1 kinase [59]–[63] to confer ∼100x activation [64]. In LKB1 kinase-deficient murine cells, AMPK is not activated by phenformin, a metformin-related biguanide [65], supporting an essential role for LKB1 kinase in drug action. We therefore tested whether *C. elegans* LKB1 homolog PAR-4 is required for healthspan benefits of metformin in the nematode model. We treated two independent *lkb1/par-4* temperature-sensitive mutants [66], [67] with increasing metformin concentrations and monitored viability during adulthood. Metformin treatment did not confer healthspan benefits in the *lkb1/par-4* mutants (Fig. 3B; see Table S1F for data from three trials using the *par-4(it47)* strain and four trials with *par-4(it57)* animals). Interestingly, as we found to be the case when AMPK activity is absent, 50 mM metformin treatment can be detrimental when LKB1 is disrupted (Table S1F). We conclude that *lkb1/par-4* is required for health-promoting action of metformin in *C. elegans* and, based on previous studies in *C. elegans* [68] and mammals [59]–[61] identifying LKB1 as the activating kinase of AMPK, suggest that LKB1 acts upstream of AAK-2 to transduce metformin healthspan benefits.

### Metformin Requires the SKN-1 Transcription Factor to Extend Median Lifespan

**Figure 4:**
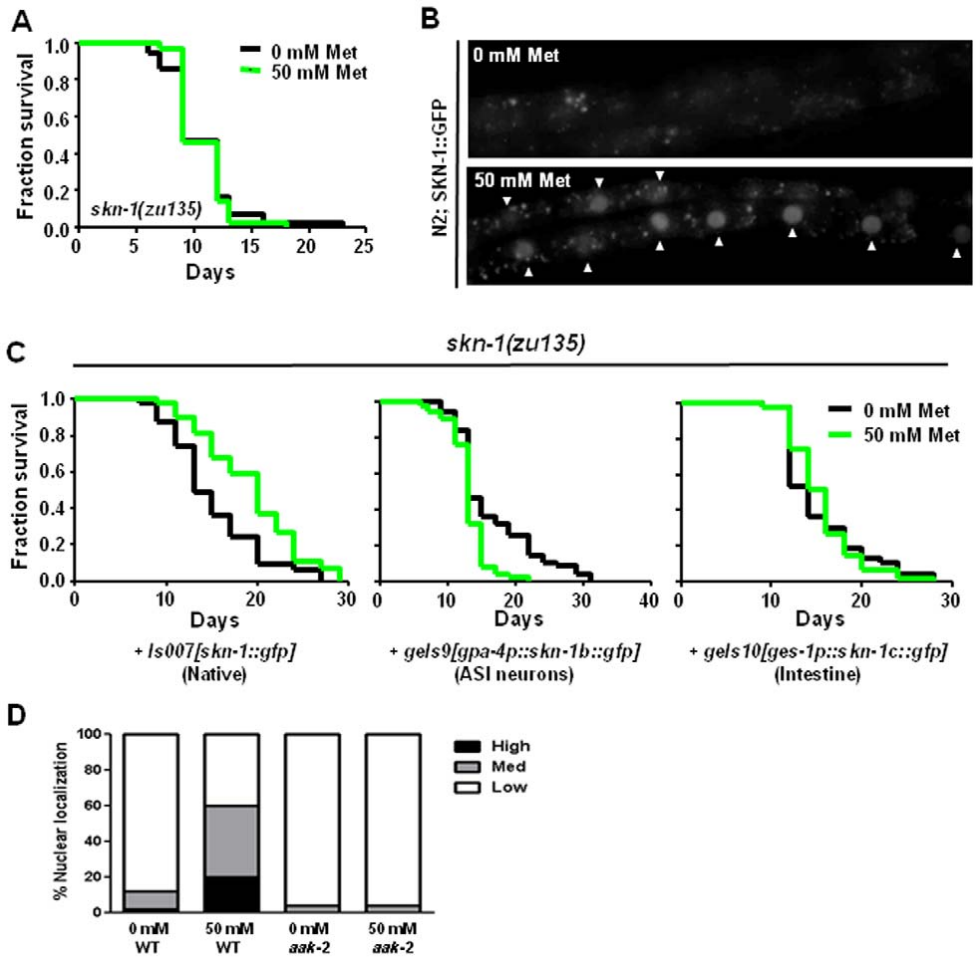
Metformin requires transcription factor SKN-1 to increase median lifespan, via a mechanism that acts in both ASI neurons and the intestine. **A.** Survival curves of *skn-1(zu135)* mutants raised on 0 mM and 50 mM metformin plates at 20°C. The median survival of animals raised on both 0 mM and 50 mM metformin plates is 9 days, and the survival curves are not significantly different. Pooled data from a total of two experiments show no change in median lifespan or survival curves (see Table S1G). We conclude that *skn-1* is required for metformin's effect on median lifespan. **B.** SKN-1 localization in L4-stage wild-type animals expressing SKN-1::GFP grown on 0 mM and 50 mM metformin plates at 20°C. SKN-1 is present constitutively in the nuclei of ASI neurons [69] where it mediates the increased longevity response under dietary restriction. Under oxidative stress and with reduced insulin signaling, SKN-1 accumulates in intestinal nuclei leading to SKN-1 target gene expression [35], [69], [71]. Whereas SKN-1::GFP is not apparent in intestinal nuclei of animals raised on 0 mM metformin plates (top panel), SKN-1::GFP accumulates in intestinal nuclei (arrowheads, bottom panel) of animals exposed to 50 mM metformin. **C.** Survival curves of *skn-1(zu135)* animals expressing *skn-1* isoforms from either the native *skn-1* promoter (*Is007[skn-1::gfp]*), an ASI neuron-specific promoter (*geIs9[gpa4p::skn-1b::gfp]*), or an intestine-specific promoter (*geIs10[ges-1p::skn-1c::gfp]*), and raised on 0 mM or 50 mM metformin plates at 20°C. While expressing *skn-1::gfp* from the native *skn-1* promoter rescues the inability of metformin to increase the lifespan of *skn-1(zu135)* mutants (see **A**) (median lifespan of *skn-1(zu135)*; *Is007[skn-1::gfp]* animals raised on 0 mM or 50 mM metformin is 13 and 20 days, respectively, and metformin treatment shifts the survival curve or these animals significantly to the right, *P = *0.0008 by the Log-rank test), expressing *skn-1* only in the ASI neurons or only in the intestine does not. In fact, metformin has significantly detrimental effects in *skn-1(zu135)* mutants that express *skn-1::gfp* only in the ASI neurons (mean lifespan for these animals raised on 0 mM or 50 mM metformin is 18 and 13 days, respectively, and the surivival curve with metformin treatment is shifted significantly to the left, *P*<0.0001 by the Log-rank test). We performed each of these lifespan assays a total of three times with similar results, see Table S1G. Pooled data for *skn-1(zu135)* animals expressing *skn-1::gfp* from the native *skn-1* promoter show a median lifespan increase from 16 days for animals on 0 mM metformin to 20 days for animals on 50 mM metformin (a 25% increase), with a significantly right-shifted survival curve (*P*<0.0001 by the Log-rank test, Table S1G). Pooled data for *skn-1(zu135)* mutants expressing *skn-1::gfp* only in the ASI neurons show a median lifespan decrease from 16 days for animals on 0 mM metformin to 13 days for animals on 50 mM metformin, with a significantly left-shifted survival curve (*P*<0.0001 by the Log-rank test, Table S1G). Note that in these experiments we cannot rule out that the tissue-specific expression of *skn-1* driven by heterologous promoters provides the normally appropriate level of expression, and thus the negative effects in ASI and intestine must be evaluated with cautious attention to this caveat. **D.** Quantification of SKN-1::GFP nuclear localization. Wild-type animals expressing SKN-1::GFP display significantly higher incidences of SKN-1::GFP accumulation in intestinal nuclei vs. controls when exposed to 50 mM metformin (*P*<0.0001 by the Chi-square test). Strikingly, AMPK is required for this effect: 50 mM metformin does not induce nuclear SKN-1::GFP accumulation in the *aak-2(ok524)* mutant background. “Low” indicates very little or no SKN-1::GFP localization to intestinal nuclei; “Medium” indicates strong SKN-1::GFP localization to nuclei in the anterior and/or posterior of the intestine; “High” indicates strong SKN-1::GFP accumulation in nuclei throughout the intestine. Although 10 mM NaN_3_ did not trigger SKN-1::GFP nuclear localization on its own, we note that NaN_3_ is an oxidative stressor that has been shown to induce nuclear SKN-1::GFP accumulation at high concentrations [96]. To confirm that 50 mM metformin can induce SKN-1::GFP nuclear accumulation in the absence of NaN_3_, we performed these experiments with N2 *Is007[skn-1::gfp;rol-6dm]* animals raised from eggs on 0 mM and 50 mM metformin and observed in liquid M9 media. We again found that 50 mM metformin significantly increases SKN-1::GFP nuclear accumulation under these conditions (Fig. S3A).

How metformin-activated AMPK exerts downstream effects to confer healthspan benefit is not known. Given our evidence that metformin induces DR-like physiology in *C. elegans* (Fig. 2B–E; Fig. S1), we sought to evaluate how AMPK activation and dietary restriction metabolism might be linked by testing known DR modulators. The *C. elegans* SKN-1 transcription factor, functionally and structurally related to mammalian Nrf transcription factors, contributes to lifespan extension via both DR and oxidative stress response pathways [35], [69], [70]. To investigate whether SKN-1 is required for metformin to increase median lifespan, we tested for metformin effects on the *skn-1(zu135)* mutant, which encodes truncated SKN-1 isoforms lacking DNA binding domains [35], [70]. We find that metformin no longer confers median lifespan benefit in the *skn-1(zu135)* background (Fig. 4A; similar results in a repeat of this experiment in Table S1G), suggesting that *skn-1* plays an essential role in metformin's positive effects.

As independent confirmation that metformin promotes SKN-1 activation, we tested for SKN-1::GFP nuclear translocation in intestine in response to metformin. SKN-1::GFP accumulates in intestinal nuclei under conditions of oxidative stress or low insulin signaling [35], [69], [71]. Consistent with metformin activation of intestinal SKN-1 to increase healthspan, we found that metformin treatment results in an increase in the frequency of nuclear SKN-1::GFP in the intestine (Fig. 4B and Fig. S3A; SKN-1 localizes constitutively to the nuclei of the ASI neurons so similar study in these neurons is not possible [69]). We also found that metformin treatment induces the expression of SKN-1 target *gst-4*, which encodes glutathione transferase-4 and is involved in the oxidative stress response in the intestine (Fig. S3B) [72], [73]. We conclude that metformin stimulates SKN-1 nuclear translocation in the intestine to promote SKN-1-dependent transcription. Our data reveal SKN-1 as a critical executor of metformin-induced healthspan extension in *C. elegans*. To the best of our knowledge, this is the first implication of SKN-1 in metformin-mediated benefits. Our data raise the possibility that mammalian Nrf transcription factors may play similar roles.

### Metformin Must Activate Both SKN-1-Dependent Pathways of DR Activation and Oxidative Stress Resistance to Extend *C. Elegans* Healthspan

*skn-1* is expressed in ASI neurons and intestine but appears to affect different pathways in these cell types to influence either DR or oxidative stress resistance lifespan extension [35], [69], [71]. *skn-1* expressed in only two ASI neurons in an otherwise *skn-1* mutant background can promote the long lifespan under dietary restriction, whereas *skn-1* expressed in the intestine contributes oxidative stress resistance and the increased longevity resulting from reduced insulin signaling [35], [69], [71].

To determine whether SKN-1 acts in either the ASI neurons or the intestine to mediate metformin-induced healthspan extension, we tested genetic mosaics that express tissue-appropriate isoforms of *skn-1* from the *gpa-4* promoter specific to ASI neurons [74] or the *ges-1* promoter specific to the intestine [75] in the *skn-1(zu135)* background (Fig. 4C). We find that metformin does not increase the healthspan of *skn-1(zu135)* mutants that express *skn-1* in only the ASI neurons or that express *skn-1* only in the intestine. However, we find that metformin does significantly increase the median lifespan of *skn-1(zu135)* mutants expressing *skn-1* from its native promoter, i.e., in *both* the ASI neurons and the intestine (20 days for 50 mM metformin treatment vs. 13 days for the control, a 54% increase; *P* = 0.0008 by the Log-rank test). We observed similar results in two additional trials of each of these experiments (Table S1G).

Taken together, our results suggest that SKN-1 must function in both the ASI neurons and the intestine for metformin to convey healthspan benefits in *C. elegans*.

### AMPK Acts Upstream to Activate SKN-1 Transcriptional Activity

Given that metformin effects require both AMPK and SKN-1, we wondered whether they act sequentially in the same pathway. To begin to evaluate their relative order of action we tested whether the nuclear localization of SKN-1::GFP depends on AMPK activity by constructing SKN-1::GFP reporter lines that included *aak-2(ok524)* and monitoring GFP localization with and without 50 mM metformin. We found that metformin-induced SKN-1::GFP nuclear localization is strongly AMPK-dependent: in the *aak-2(ok524)* mutant background, metformin treatment no longer induces nuclear SKN-1::GFP accumulation (Fig. 4D). These data suggest that AMPK acts upstream of SKN-1 to transduce metformin's effects, at least in intestine.

**Figure 5:**
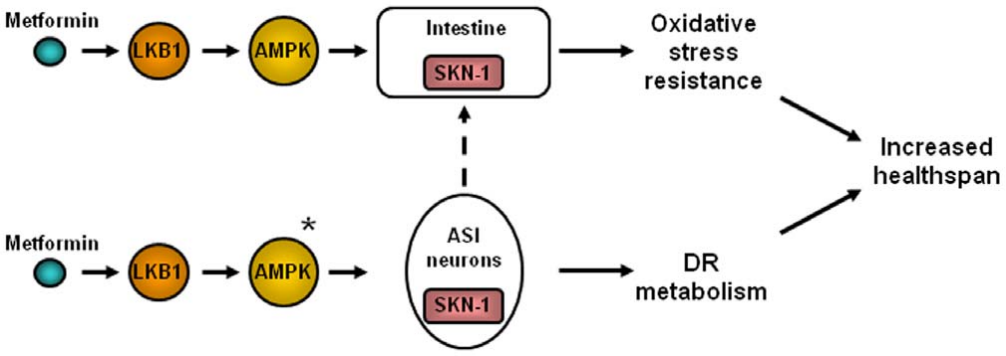
Summary model of metformin action on *C. elegans* healthspan. Metformin activates SKN-1 to trigger DR-like metabolism and intestinal anti-oxidant gene expression. SKN-1 is needed in both the two ASI neurons and in the intestine for metformin to be beneficial. Since SKN-1 in the ASI neurons has been shown previously to be needed for DR lifespan extension [35], it seems probable that the ASIs play a role in the DR-like metabolism induced by metformin. LKB1 and AMPK are needed for metformin health benefits and AMPK acts upstream of intestinal SKN-1 for metformin benefits. *The relative order of action of SKN- and AMPK has not yet been experimentally addressed for ASI neurons. Dotted line—it is possible that signaling in the neurons could activate SKN-1 in intestine.

We suggest a model in which metformin activates both DR metabolism (presumably through two ASI neurons), as well as oxidative stress resistance mechanisms—this combination confers metformin-dependent healthspan extension (Fig. 5).

## Discussion

Here we describe the first genetic dissection of molecular requirements for metformin-induced healthspan extension in a whole animal context. We report that metformin extends *C. elegans* median lifespan via a mechanism that requires the cellular energy sensor AMPK, its upstream activating kinase LKB1, and the downstream DR/oxidative stress responsive transcription factor SKN-1, but is independent of the insulin signaling pathway components that modulate longevity. Our data on metformin outcomes are consistent with the interpretation that metformin acts, at least in part, as a dietary restriction mimetic in *C. elegans* and suggest that this activity is executed via a mechanism that is conserved from nematodes to humans. Implications of these findings could influence development of plausible anti-aging therapies based on a drug currently used in the clinic.

### Metformin Prolongs Youthfulness and Extends the period of Healthy Maintenance in *C. elegans* Cultures via a Mechanism That Induces DR Characteristics

In mammals, biochemical and physiological studies have suggested that metformin acts as a DR mimetic [2], [4], [5], [18], but until recently [18] genetic data in support of this activity have been lacking. Our results support that metformin induces a DR-like state in well-fed *C. elegans* in that it does not affect the pharyngeal pumping function required for feeding, but it does induce multiple features of DR: 1) extension of median lifespan; 2) DR-specific age pigment properties [50]; 3) low fat stores; and 4) reduced fecundity. Furthermore, metformin is unable to extend median lifespan of the feeding-compromised *eat-2* mutant; instead, metformin doses that normally benefit wild-type actually are deleterious to *eat-2*, possibly by inducing a starvation-like state from excessive metabolic shutdown. Both of these responses are consistent with metformin inducing a DR-like state that is exacerbated in the *eat-2* mutant. Finally, we show that metformin requires at least one essential DR transcription factor, SKN-1 [35], to confer healthspan benefit. Taken together, our data strongly support that metformin acts in *C. elegans* at least in part by shifting metabolism to a DR-like state and, importantly, suggest that the capacity for metformin to do so has been maintained from invertebrates to humans.

One seemingly paradoxical observation in relation to classic DR outcomes is that we did not find that maximum lifespan is extended by metformin treatment as is typical of DR across species [6]–[13]. One possible reason for this is that the 50 mM treatment that we selected for most analyses might actually be suboptimal for healthspan benefits, corresponding to a dose that shifts animals partially into a zone of metabolism in which starvation consequences begin to negatively impact viability over the longterm. Alternatively, increased ROS burden associated with metformin treatment might not be effectively managed later in life (see further discussion below). Known side effects of biguanides in humans include deleterious lactic acidosis in treated diabetics [76], and thus it is also possible that acidosis limits healthspan benefits.

### AMPK And LKB1 Are Required for Metformin to Positively Impact Healthspan

In mammalian models, metformin and other biguanides activate AMPK in reactions needed for some metformin-associated outcomes [20], [21], [65]. In *C. elegans,* both an *aak-2* deletion mutant and a catalytically inactive *aak-2* mutant are defective in metformin-induced median lifespan extension. Requirement for AMPK activity for healthspan benefits may be a feature of metformin action conserved across species.

AMPK has been implicated in *C. elegans* lifespan regulation in multiple pathways [55], [56], [77], including DR [34] and the lifespan-promoting activities of limiting glycolytic flux by 2-deoxyglucose treatment [78], which features some aspects of DR. Interestingly, however, the requirement for AMPK in *C. elegans* DR appears dependent upon the approach to food limitation–extending lifespan by directly inducing DR through bacterial dilution on plates requires AMPK activity in *C. elegans* [34] but *aak-2* has been reported to be dispensable for the long lifespan of DR-constitutive *eat-2* animals [56]. Different DR models in *C. elegans* may thus have distinct genetic requirements, and multiple DR pathways that extend lifespan may co-exist. Indeed, our observation that functional *aak-2* is required for metformin to reduce overall fat levels, but not age pigment accumulation (Fig. S2B and Fig.S2C), suggests that metformin may be signaling through different pathways to trigger DR-like physiology.

How is AMPK likely to promote DR-like metabolism? In mammals, there are several known AMPK targets that modulate metabolic pathways including those responsible for fatty acid, protein, and cholesterol synthesis, as well as those that mediate glucose transport and glycolysis in liver and muscle [77], [79], [80]. The power of the worm model is that *C. elegans* proteins orthologous to mammalian metformin targets like glycogen synthase (which is inhibited in mammals by AMPK [81], [82]), TOR kinase complex TORC2 (inhibited by AMPK from promoting gluconeogenesis by inducing PEPCK and glucose-6-phosphatase transcription [83]), PFK-2/FBPase-2 (a target of AMPK that regulates glycolytic/gluconeogenic flux [84]), and hormone-sensitive lipase (LIPE) (which mobilizes free fatty acids from stored triglycerides [85]) can be manipulated to test relative roles in the various measures of healthspan.

In terms of upstream activation of AMPK, we report that kinase LKB1 is also essential for metformin benefit. Given that LKB1 is the identified upstream kinase for *aak-2* [68], the simplest model is that metformin promotes activation of LKB1, which phosphorylates and activates AMPK to promote healthspan. That this activation pathway appears operative in mammalian DR and metformin action [19]–[21] is further evidence for metformin acting via a mechanism conserved from nematodes to humans. Exactly how LKB1 is activated by metformin is a question that remains to be addressed.

### Metformin Requires the Functionally Conserved SKN-1 Transcription Factor in Both the ASI Neurons and the Intestine to Increase Healthspan

*C. elegans* SKN-1 is needed for endodermal development, controls the phase 2 detoxification response that defends against oxidative stress, and is required for the oxidative stress resistance and longevity phenotypes associated with reduced insulin signaling [69]–[71]. SKN-1 also functions in two ASI neurons to promote lifespan extension under DR conditions [35]. Here we report for the first time that SKN-1 is required for metformin to extend median lifespan in *C. elegans*, and provide evidence that metformin activates both DR and oxidative stress resistance mechanisms to promote healthspan.

The requirement for SKN-1 activity in only ASI neurons for DR benefits [35] suggests that ASI normally senses food limitation and signals to coordinate metabolism in the rest of the body tissues. That metformin induces a DR-like state via a SKN-1-dependent mechanism raises the possibility that critical metformin action in DR induction might be similarly targeted to these two regulatory neurons in *C. elegans*. Indeed, we find that SKN-1 action in ASI neurons is necessary for the healthspan benefits of metformin, although SKN-1 function in the ASI neurons is not sufficient for metformin-mediated healthspan extension.

SKN-1 also acts in the *C. elegans* intestine to promote longevity in response to low insulin pathway signaling and oxidative stress conditions [35], [69], [71]. We have shown that metformin activates AMPK to promote SKN-1 nuclear translocation and expression of SKN-1 target *gst-4* in intestine. Intestinal SKN-1 activity is necessary but not sufficient for metformin healthspan benefits. Because we observed healthspan benefits only under conditions in which *skn-1* is expressed from its native promoter (characterized to be active in intestine and ASI neurons [35]), we infer that metformin requires SKN-1 activities in both ASI neurons and intestinal SKN-1 to increase healthspan. We note, however, that this conclusion rests on the assumption that the tissue-specific transgene expression we tested (functional in ref [35]) provides the expression levels appropriate for rescuing SKN-1 functions in these tissues.

How does metformin activate SKN-1? Metformin has been shown to increase ROS production in mammalian cells [86]. In *C. elegans*, DR has been associated with sensitivity to ROS stressors ([87] and Fig. S4), low glucose levels have been shown to promote ROS formation in [78], and LKB1/PAR-4 has been shown to be the upstream kinase of AAK-2 in a pathway needed for oxidative stress resistance [68], implicating ROS in pathways relevant to the metformin model. We therefore speculate that metformin may raise ROS levels to activate intestinal SKN-1, where it functions to defend against oxidative stress and promote longevity. Such a model is consistent with our observation that metformin treatment is actually detrimental to the animals expressing SKN-1 only in the ASI neurons and not in the intestine (Fig. 4C)–intestinal SKN-1 may promote organism healthspan by counteracting some of the effects of metformin-induced SKN-1 signaling in ASI neurons.

SKN-1 is orthologous to the mammalian Nrf transcription factors (Nrf1 and Nrf2) that activate genes encoding phase 2 detoxification enzymes [88], [89]. Notably, Nrf2 defends against age-related diseases such as neurodegeneration, chronic inflammation, and cancer [90], [91]. Our results highlight the potential of Nrf transcription factors to play required roles in health-promoting effects of metformin in mammals.

### A Genetic Tool for Evaluating Healthspan Therapies

In humans, metformin increases survival rates in diabetic and cardiovascular disease patients [1], [2], but healthspan impact on non-diseased individuals has not been established. In *C. elegans*, metformin treatment extends median lifespan in normal wild type animals as well as in mutants with disrupted insulin signaling pathways, supporting potential application of metformin as a prophylatic anti-aging strategy in healthy individuals. Because the nematode model exhibits multiple commonalities associated with metformin treatment in mammals, it appears that the basic mechanism of metformin action involves conserved metabolic relationships. We suggest that the *C. elegans* model is therefore well suited to detailed elucidation of the physiological mechanism of metformin action and may be particularly useful for screening for chemically modified biguanides that have increased efficacy in healthspan promotion but lack deleterious side effects associated with metformin.

Overall, our results support that metformin acts through LKB1, AMPK, and SKN-1 in a conserved biochemical mechanism to engage DR metabolism and anti-oxidant defenses to extend healthspan in *C. elegans*, a conclusion that holds significant implications for development of anti-aging therapies.

## Materials and Methods

### *C. elegans* Cultures

The following strains used in this study were obtained from the *Caenorhabditis* Genetics Center (CGC, University of Minnesota): wild-type Bristol N2, TJ1052 *age-1(hx546)*, CB1370 *daf-2(e1370)*, GR1307 *daf-16(mgDf50)*, DA1116 *eat-2(ad1116)*, RB754 *aak-2(ok524)*, MR507 *aak-2(rr48)*, KK184 *par-4(it47)*, KK300 *par-4(it57)*, CL2166 N2 *dvIs19[pAF15(gst-4::GFP::NLS)]* and EU31 *skn-1(zu135)/nT1[unc-?(n754) let-?]*. LD001 N2 *Is007[skn-1::gpf;rol-6dm]* was generated by J. H. An and T. K. Blackwell. The *skn-1* strains used for mosaic analyses, LG333 *skn-1(zu135);Is007[skn-1::gfp;rol-6dm]*, LG348 *skn-1(zu135)/nT1[qIs51];geIs9[gpa-4p::skn-1b::gfp; rol-6(su1006)]*, and LG357 *skn-1(zu135)/nT1[qIs51];geIs10[ges-1p::skn-1c::gfp; rol-6(su1006)]*, were kindly provided by L. Guarente and N. Bishop. All strains were grown and maintained on NGM plates seeded with *Escherichia coli* OP50 [92]. All experiments were performed at 20°C except for lifespan assays with *daf-2(e1370), par-4(it47)*, and *par-4(it57)* (see below). Metformin (Aldrich, Catalog # D15,095-9) was added directly to the NGM agar media to a final concentration of 1, 10, 50, or 100 mM from a 1 M aqueous stock. Note that *C. elegans* is often fairly resistant to drug uptake and doses in the worm are expected to be much lower than plate concentrations.

### Lifespan Assays

Lifespan analyses were performed in the same manner for all strains, except for experiments with *daf-2(e1370), par-4(it47)*, and *par-4(it57)*. *daf-2(e1370)* L2 larvae enter diapause at restrictive temperatures [25], [93] and thus *daf-2(e1370)* animals were maintained at 15°C and shifted to 20°C on the first day of adulthood during lifespan assays. The *par-4* strains are temperature-sensitive maternal effect lethal [66], [67], and were maintained at 15°C and shifted to 25°C at the L4 stage for lifespan studies. The *daf-2(e1370)* and *par-4* animals were placed on metformin-containing plates on the day of temperature shift. For each experiment, except where otherwise noted, the lifespan of 60 animals was measured in each trial. For each concentration of metformin tested, we placed 15 L4-stage larvae on 3 plates with 5 animals per plate and allowed these to develop to adulthood and then lay eggs over 24 hours. These parental animals were then removed from the plates. 48 hours later, 60 (day 2) L4 larvae were transferred to fresh plates. These animals were transferred to fresh plates every day during the progeny production period, and then every other day thereafter. Animals that did not move when gently prodded were scored as dead. Animals that crawled off the plate or died from vulva bursting or internal hatching were not included in lifespan counts.

### Locomotion Assays

Animals were raised from eggs similar to the lifespan assays. On days 5, 10, and 15 of life, 30 individuals from the control or experimental plates were measured for body bend rate in liquid. Briefly, 10 animals were placed in 20 µl M9 buffer on a glass slide and filmed for 30 seconds using a Qimaging Rotera-XR digital camera attached to a dissecting microscope and Streampix imaging software (ver. 3.17.2, NorPix). Body bends were counted by reviewing each frame of the 30 second film. Results are the averages of three independent trials.

### Age Pigment Fluorescence Spectroscopy

Age pigment fluorescence intensity was measured as described [50]. Wild-type animals were raised from eggs on plates as in the lifespan assays until day 5 of life. On day 5, 50 animals were transferred to 50 µl 10 mM NaN_3_ solution in a single well of a 96-well white FluoroNunc plate (Nalge Nunc Int'l). The animals were scanned using an *in vivo* spectrofluorimeter (Fluorolog-3, Jobin Yvon Inc., Edison NJ). Peak age pigment fluorescence intensity was determined by scanning through a range of excitation wavelengths from 280–410 nm and an emission wavelength of 430 nm. DataMax data acquisition software (v. 2.20, Jobin Yvon Inc.) and Grams/32 data manipulation software (v. 4.14, Galactic Industries Corp.) were used to process the emission data. Scores are the average age pigment fluorescence intensity levels of three independent trials.

### Fecundity Assays

Wild-type animals were raised from eggs similar to the lifespan assays. Animals were transferred to fresh plates every day during the progeny production period, and the original plates were left at 20°C for 24 hours to allow viable eggs to hatch and were then stored at 4°C to be scored at the end of the experiment. Data shown represent the average of three independent trials.

### Nile Red Staining and Fluorescence Spectroscopy

Wild-type L4 animals were placed on plates and allowed to develop for 2 days. For lipid staining, these egg-laying parental animals were transferred to plates layered with 120 µl of a 500 µg/mL Nile Red (Molecular Probes N-1142) [53], [54] stock in acetone or control plates containing no Nile Red. Gravid adults were allowed to lay eggs for several hours, then removed. 5 days later, progeny were scanned for Nile Red fluorescence similar to as described for age pigments. Nile Red fluorescence intensity was read at an emission wavelength of 658 nm and an excitation wavelength of 578 nm [53]. There was no detectable fluorescence at these excitation and emission wavelengths in worms raised on plates containing no Nile Red dye. Data represent the average of three independent experiments.

### Pharyngeal Pumping Assays

Wild-type animals were raised from eggs on plates similar to the lifespan assays. On day 4 of life, the pharyngeal pumping rates of 10 individuals were measured by transferring single animals to an unseeded plate and scoring pharyngeal pumping under a dissecting microscope for 30 seconds. Data represent the averages of three separate experiments.

### Nuclear SKN-1::GFP Quantitation

N2 *Is007[skn-1::gfp;rol-6dm]* or *aak-2(ok524)*;*Is007[skn-1::gfp;rol-6dm]* animals were raised from eggs as in the lifespan assays. L4 animals were placed in 10 mM NaN_3_ or M9 liquid media and observed under a 40x objective lens using a Zeiss Axioplan 2 microscope equipped with an X-cite Series 120 (EXPO Photonic Solution, Inc.) fluorescence illuminator. Micrographs were obtained using an Optronics digital microscope camera and Magnafire processing software. 50 animals were observed for each experimental condition.

### Statistical Analyses

Log-rank (Mantel-Cox) tests, Gehan-Breslow-Wilcoxon tests, and unpaired t tests were performed using GraphPad Prism version 5.00 for Windows (GraphPad Software, San Diego California).

## Supporting Information

Figure S1 Metformin triggers the dietary restriction (DR)-specific fluorescent profile. Age pigment fluorescence measurements in wild-type animals raised on 0 mM and 100 mM metformin plates. As in Fig. 2B, we measured age pigment fluorescence in five-day-old nematodes. In the trial presented here, 100 mM metformin decreased age pigment fluorescence levels as compared to the 0 mM control (86413 a.u. versus 148734 a.u., respectively; a.u. = arbitrary units). In addition, 100 mM metformin induced a shift in the excitation wavelength corresponding to the maximum age pigment fluorescence intensity (ExMax shift, 331 nm for 100 mM metformin versus 339 nm for the 0 mM control). Since 100 mM metformin did not extend mid-life viability well (possibly due to starvation or excessive DR signaling), we chose to work with lower doses of the drug.
(0.07 MB TIF)

Figure S2 Metformin has detrimental effects on healthspan if AMPK is mutant. A. Swimming rates for aak-2(ok524) and aak-2(rr48) AMPK mutants raised from eggs on plates containing 0 mM and 50 mM metformin at 20°C. Swimming was recorded on days 5 and 10 of life as in Fig. 1B. In both strains, 50 mM metformin significantly decreases swimming rates on both day 5 and day 10 of life (P = 0.0002 and P<0.0001 for aak-2(ok524) on days 5 and 10, respectively; P = 0.0272 and P<0.0001 for aak-2(rr48) on days 5 and 10, respectively), indicating that metformin decreases locomotory healthspan when AMPK signaling is disrupted. These data represent the averages of three independent trials for each experiment. B. Nile Red levels of aak-2(ok524) mutants raised on 0, 1, 10, and 50 mM metformin were measured on day 5 of life. Data from three independent trials show that metformin had no significant effect on the levels of Nile Red fluorescence at any of the tested concentrations. C. Exposure to 50 mM metformin significantly reduced levels of age pigments (P = 0.0327). These results indicate that functional AMPK is required for metformin to reduce fat levels, but not age pigment accumulation, in *Caenorhabditis elegans*. These observations suggest that metformin signals through different pathways to influence healthspan.
(0.10 MB TIF)

Figure S3 Metformin induces intestinal nuclear SKN-1::GFP accumulation and the expression of SKN-1 target gene reporter, pgst-4GFP::NLS. A. N2 Is7[skn-1::gfp;rol-6dm] animals were raised from eggs on 0 mM or 50 mM plates to the L4 stage, and then placed in liquid M9 media for quantification of intestinal nuclear SKN-1::GFP accumulation. Animals exposed to 50 mM metformin had significantly higher levels of nuclear SKN-1::GFP accumulation versus controls (P<0.0001 by the Chi-square test). 90 animals were examined for the 0 mM metformin group and 93 animals were observed for the 50 mM metformin group. “Low” indicates very little or no SKN-1::GFP localization to intestinal nuclei; “Medium” indicates strong SKN-1::GFP localization to nuclei in the anterior and/or posterior of the intestine; “High” indicates strong SKN-1::GFP accumulation in nuclei throughout the entire intestine. B. Animals expressing a transcriptional fusion reporter of the SKN-1 target gst-4 (dvIs19[pAF15(gst-4::GFP::NLS)]) [72] were raised on 0 and 50 mM metformin, and GFP fluorescence intensity was measured on day 5 of life using a spectrofluorimeter. Animals exposed to 50 mM metformin had significantly higher GFP fluorescence levels versus controls (P = 0.0249), indicating the induction of gst-4 expression by metformin. The results of two independent trials are shown here.
(0.07 MB TIF)

Figure S4 Dietary restriction (DR)-constitutive eat-2(ad1116) mutants are sensitive to oxidative stress. The survival rates of wild-type N2, the mev-1(kn1) mitochondrial cytochrome b subunit mutant, and the DR-constitutive eat-2(ad1116) mutants were measured with exposure to 100 mM of the oxidative stressor paraquat on day 5 of life. Fifty animals per strain were used per trial, and the pooled results of two independent trials are shown here. mev-1(kn1) mutants are sensitive to paraquat, and showed significantly reduced survival rates when exposed to 100 mM paraquat versus N2 (P<0.0001, Log-rank test). eat-2(ad1116) mutants were even more sensitive to paraquat than mev-1(kn1) animals, and showed significantly reduced survival rates on 100 mM paraquat as compared to both N2 and mev-1(kn1) (P<0.0001 for both, Log-rank test).
(0.06 MB TIF)

Table S1 Lifespan data for all metformin trials.
(0.27 MB DOC)
