# Supplementary figures and images for: Metformin Induces a Dietary Restriction–Like State and the Oxidative Stress Response to Extend C. elegans Healthspan via AMPK, LKB1, and SKN-1
Source: PLoS One. 2010 Jan 18;5(1):e8758. doi: 10.1371/journal.pone.0008758 (PMC2807458; doi:10.1371/journal.pone.0008758)

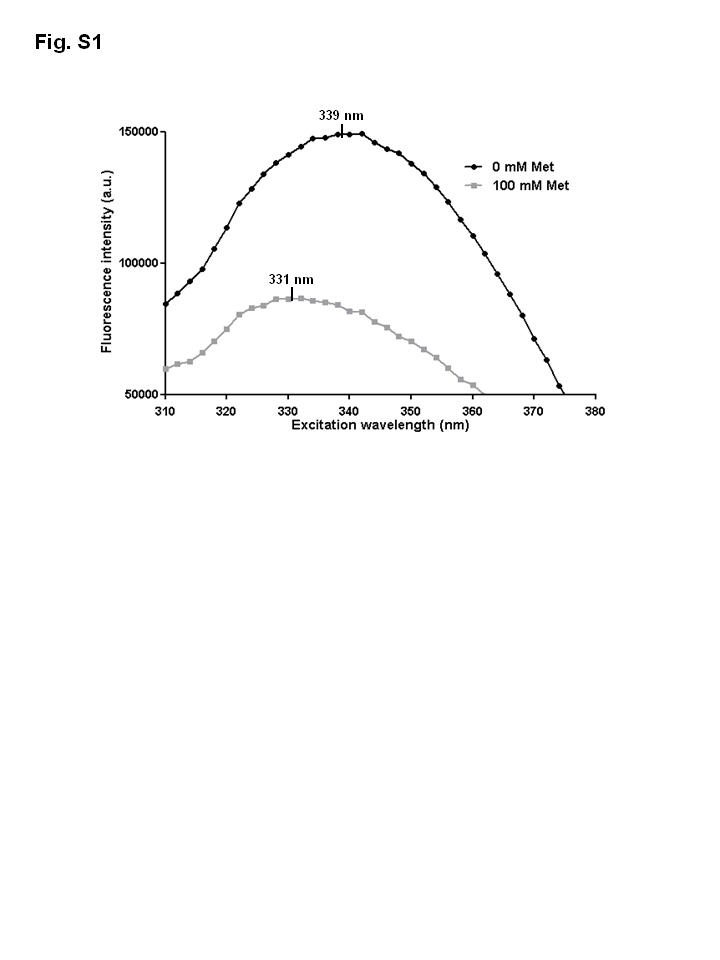

Supplement: Figure S1 — Metformin triggers the dietary restriction (DR)-specific fluorescent profile. Age pigment fluorescence measurements in wild-type animals raised on 0 mM and 100 mM metformin plates. As in Fig. 2B, we measured age pigment fluorescence in five-day-old nematodes. In the trial presented here, 100 mM metformin decreased age pigment fluorescence levels as compared to the 0 mM control (86413 a.u. versus 148734 a.u., respectively; a.u. = arbitrary units). In addition, 100 mM metformin induced a shift in the excitation wavelength corresponding to the maximum age pigment fluorescence intensity (ExMax shift, 331 nm for 100 mM metformin versus 339 nm for the 0 mM control). Since 100 mM metformin did not extend mid-life viability well (possibly due to starvation or excessive DR signaling), we chose to work with lower doses of the drug. (0.07 MB TIF) [file pone.0008758.s001.tif]

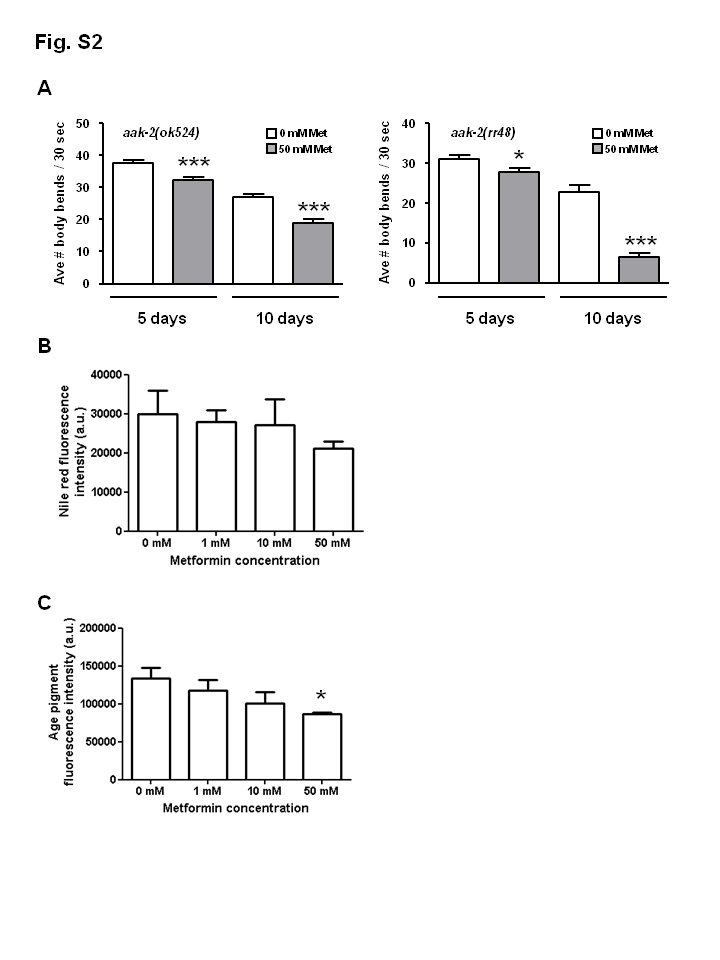

Supplement: Figure S2 — Metformin has detrimental effects on healthspan if AMPK is mutant. A. Swimming rates for aak-2(ok524) and aak-2(rr48) AMPK mutants raised from eggs on plates containing 0 mM and 50 mM metformin at 20°C. Swimming was recorded on days 5 and 10 of life as in Fig. 1B. In both strains, 50 mM metformin significantly decreases swimming rates on both day 5 and day 10 of life (P = 0.0002 and P<0.0001 for aak-2(ok524) on days 5 and 10, respectively; P = 0.0272 and P<0.0001 for aak-2(rr48) on days 5 and 10, respectively), indicating that metformin decreases locomotory healthspan when AMPK signaling is disrupted. These data represent the averages of three independent trials for each experiment. B. Nile Red levels of aak-2(ok524) mutants raised on 0, 1, 10, and 50 mM metformin were measured on day 5 of life. Data from three independent trials show that metformin had no significant effect on the levels of Nile Red fluorescence at any of the tested concentrations. C. Exposure to 50 mM metformin significantly reduced levels of age pigments (P = 0.0327). These results indicate that functional AMPK is required for metformin to reduce fat levels, but not age pigment accumulation, in Caenorhabditis elegans. These observations suggest that metformin signals through different pathways to influence healthspan. (0.10 MB TIF) [file pone.0008758.s002.tif]

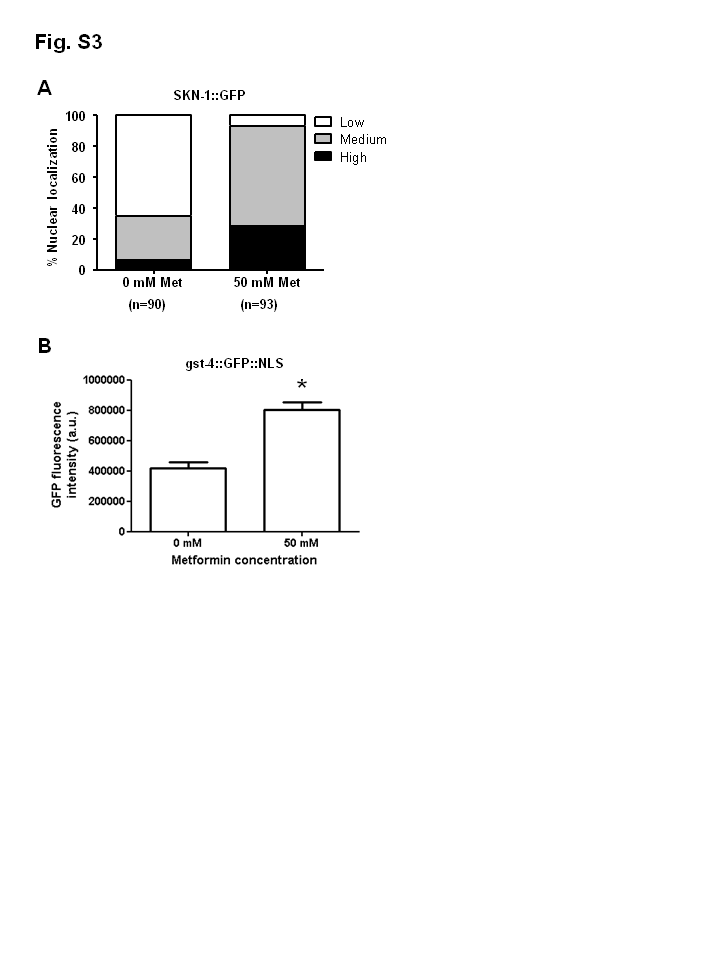

Supplement: Figure S3 — Metformin induces intestinal nuclear SKN-1::GFP accumulation and the expression of SKN-1 target gene reporter, pgst-4GFP::NLS. A. N2 Is7[skn-1::gfp;rol-6dm] animals were raised from eggs on 0 mM or 50 mM plates to the L4 stage, and then placed in liquid M9 media for quantification of intestinal nuclear SKN-1::GFP accumulation. Animals exposed to 50 mM metformin had significantly higher levels of nuclear SKN-1::GFP accumulation versus controls (P<0.0001 by the Chi-square test). 90 animals were examined for the 0 mM metformin group and 93 animals were observed for the 50 mM metformin group. “Low” indicates very little or no SKN-1::GFP localization to intestinal nuclei; “Medium” indicates strong SKN-1::GFP localization to nuclei in the anterior and/or posterior of the intestine; “High” indicates strong SKN-1::GFP accumulation in nuclei throughout the entire intestine. B. Animals expressing a transcriptional fusion reporter of the SKN-1 target gst-4 (dvIs19[pAF15(gst-4::GFP::NLS)]) [72] were raised on 0 and 50 mM metformin, and GFP fluorescence intensity was measured on day 5 of life using a spectrofluorimeter. Animals exposed to 50 mM metformin had significantly higher GFP fluorescence levels versus controls (P = 0.0249), indicating the induction of gst-4 expression by metformin. The results of two independent trials are shown here. (0.07 MB TIF) [file pone.0008758.s003.tif]

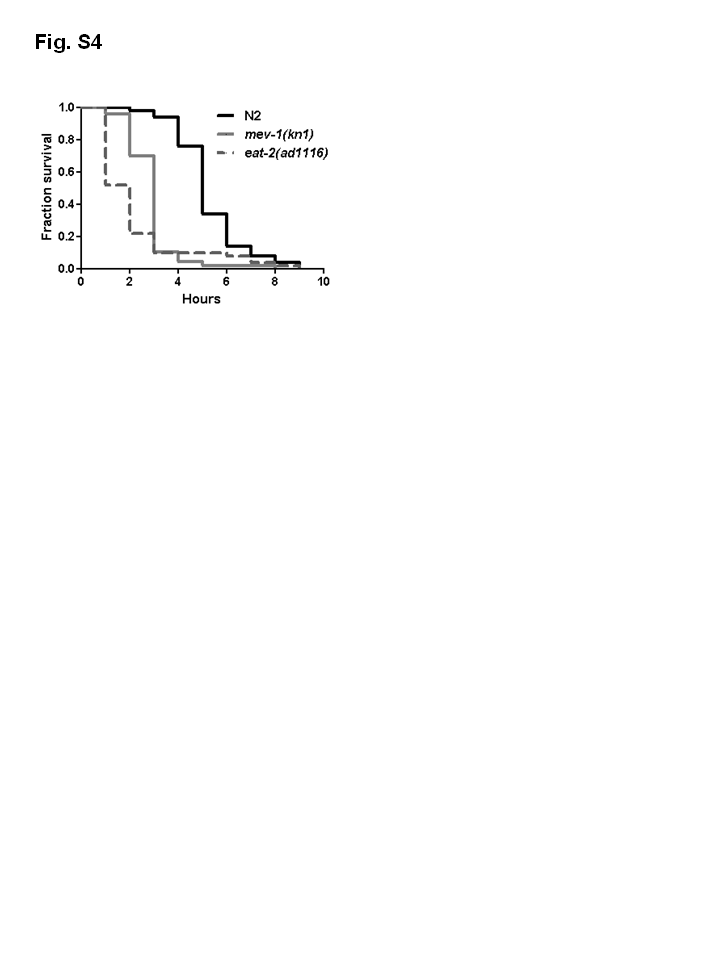

Supplement: Figure S4 — Dietary restriction (DR)-constitutive eat-2(ad1116) mutants are sensitive to oxidative stress. The survival rates of wild-type N2, the mev-1(kn1) mitochondrial cytochrome b subunit mutant, and the DR-constitutive eat-2(ad1116) mutants were measured with exposure to 100 mM of the oxidative stressor paraquat on day 5 of life. Fifty animals per strain were used per trial, and the pooled results of two independent trials are shown here. mev-1(kn1) mutants are sensitive to paraquat, and showed significantly reduced survival rates when exposed to 100 mM paraquat versus N2 (P<0.0001, Log-rank test). eat-2(ad1116) mutants were even more sensitive to paraquat than mev-1(kn1) animals, and showed significantly reduced survival rates on 100 mM paraquat as compared to both N2 and mev-1(kn1) (P<0.0001 for both, Log-rank test). (0.06 MB TIF) [file pone.0008758.s004.tif]
