# Supplementary material for: Metformin Induces a Dietary Restriction–Like State and the Oxidative Stress Response to Extend C. elegans Healthspan via AMPK, LKB1, and SKN-1
Source: PLoS One. 2010 Jan 18;5(1):e8758. doi: 10.1371/journal.pone.0008758 (PMC2807458; doi:10.1371/journal.pone.0008758)
Supplement: Table S1 — Lifespan data for all metformin trials. (0.27 MB DOC) [file pone.0008758.s005.doc]

**A. Lifespan data for wild-type animals treated with metformin**

|  | Metformin concentration | N total | N dead | N censored | Median lifespan (days) | *p* value  (log-rank) |
| --- | --- | --- | --- | --- | --- | --- |
| Trial 1 | 0 mM | 60 | 38 | 22 | 14 | N/A |
|  | 1 mM | 60 | 36 | 24 | 19 | 0.0199 |
|  | 10 mM | 60 | 36 | 24 | 17 | 0.7329 |
|  | 50 mM | 60 | 36 | 24 | 23 | 0.0006 |
| Trial 2 | 0 mM | 62 | 55 | 7 | 15 | N/A |
|  | 1 mM | 60 | 47 | 13 | 17 | 0.0724 |
|  | 10 mM | 61 | 56 | 5 | 17 | 0.3512 |
|  | 50 mM | 60 | 53 | 7 | 17 | 0.0172 |
| Trial 3 * | 0 mM | 60 | 35 | 25 | 19 | N/A |
|  | 1 mM | 60 | 38 | 22 | 19 | 0.8955 |
|  | 10 mM | 60 | 37 | 23 | 19 | 0.743 |
|  | 50 mM | 60 | 38 | 22 | 19 | 0.1882 |
| Trial 4 * | 0 mM | 62 | 39 | 23 | 17 | N/A |
|  | 1 mM | 60 | 38 | 22 | 21 | 0.0742 |
|  | 10 mM | 60 | 41 | 19 | 17 | 0.5724 |
|  | 50 mM | 59 | 39 | 20 | 15 | 0.0353 |
| Trial 5 | 0 mM | 60 | 51 | 9 | 15 | N/A |
|  | 1 mM | 60 | 51 | 9 | 15 | 0.4302 |
|  | 10 mM | 60 | 54 | 6 | 17 | 0.266 |
|  | 50 mM | 60 | 51 | 9 | 21 | <0.0001 |
| Trial 6 | 0 mM | 60 | 45 | 15 | 15 | N/A |
|  | 1 mM | 60 | 45 | 15 | 15 | 0.3519 |
|  | 10 mM | 60 | 53 | 7 | 15 | 0.3313 |
|  | 50 mM | 60 | 46 | 14 | 19 | 0.0002 |
| Trial 7 | 0 mM | 60 | 49 | 11 | 18 | N/A |
|  | 1 mM | 60 | 35 | 25 | 16 | 0.2403 |
|  | 10 mM | 60 | 47 | 13 | 16 | 0.0179 |
|  | 50 mM | 60 | 40 | 20 | 22 | 0.0523 |
| Trial 8 | 0 mM | 60 | 52 | 8 | 12 | N/A |
|  | 50 mM | 60 | 50 | 10 | 15 | 0.1482 |
| Trial 9 | 0 mM | 60 | 50 | 10 | 13 | N/A |
|  | 50 mM | 60 | 38 | 22 | 20 | 0.0001 |
| Trial 10 | 0 mM | 60 | 50 | 10 | 16 | N/A |
|  | 50 mM | 60 | 49 | 11 | 23 | <0.0001 |
| Trial 11 | 0 mM | 61 | 48 | 13 | 13 | N/A |
|  | 50 mM | 60 | 46 | 14 | 20 | <0.0001 |
| Trial 12 | 0 mM | 57 | 42 | 15 | 14 | N/A |
|  | 50 mM | 52 | 35 | 17 | 16 | 0.0100 |
| **Pooled** | **0 mM** | **722** | **554** | **168** | **15** | **N/A** |
| **wild-type** | **1 mM** | **420** | **290** | **130** | **17** | **0.0005** |
| **data** | **10 mM** | **421** | **324** | **97** | **17** | **0.1941** |
|  | **50 mM** | **711** | **521** | **190** | **19** | **<0.0001** |
| Wild-type | 0 mM | 60 | 54 | 6 | 11 | N/A |
| (25°) | 50 mM | 60 | 54 | 6 | 12 | 0.0038 |

* No increase in median lifespan was seen with 50 mM metformin treatment in these trials.

All trials were at 20°C except where otherwise indicated.

**B. Lifespan data for *daf-16(mgDf50)*** treated with metformin

|  | Metformin concentration | N total | N dead | N  censored | Median lifespan (days) | *p* value  (log-rank) |
| --- | --- | --- | --- | --- | --- | --- |
| Trial 1 | 0 mM | 100 | 65 | 35 | 15 | N/A |
|  | 1 mM | 98 | 65 | 33 | 13 | 0.4236 |
|  | 10 mM | 99 | 66 | 33 | 13 | 0.0074 |
|  | 50 mM | 100 | 65 | 35 | 19 | 0.0005 |
| Trial 2 | 0 mM | 60 | 43 | 17 | 13 | N/A |
|  | 1 mM | 60 | 43 | 17 | 13 | 0.7594 |
|  | 10 mM | 60 | 40 | 20 | 15 | 0.3131 |
|  | 50 mM | 60 | 43 | 17 | 15 | 0.045 |
| Trial 3 | 0 mM | 60 | 46 | 14 | 11 | N/A |
|  | 1 mM | 60 | 46 | 14 | 13 | 0.6716 |
|  | 10 mM | 60 | 46 | 14 | 11 | 0.7324 |
|  | 50 mM | 60 | 45 | 15 | 15 | 0.0111 |
| Trial 4 | 0 mM | 60 | 50 | 10 | 11 | N/A |
|  | 1 mM | 60 | 50 | 10 | 11 | 0.6462 |
|  | 10 mM | 60 | 50 | 10 | 13 | 0.6035 |
|  | 50 mM | 60 | 50 | 10 | 15 | 0.0006 |
| **Pooled** | **0 mM** | **280** | **204** | **76** | **13** | **N/A** |
| ***daf-16(mgDf50)*** | **1 mM** | **278** | **204** | **74** | **13** | **0.7112** |
| **data** | **10 mM** | **279** | **202** | **77** | **13** | **0.3652** |
|  | **50 mM** | **280** | **203** | **77** | **15** | **<0.0001** |

**C. Lifespan data for long-lived insulin signaling pathway mutants treated**

**with metformin**

|  | Metformin concentration | N total | N dead | N censored | Median lifespan (days) | *p* value  (log-rank) |
| --- | --- | --- | --- | --- | --- | --- |
| *age-1(hx546)* | 0 mM | 60 | 36 | 24 | 24 | N/A |
| Trial 1 | 50 mM | 60 | 45 | 15 | 31 | 0.0014 |
| *age-1(hx546)* | 0 mM | 60 | 38 | 22 | 21 | N/A |
| Trial 2 | 50 mM | 60 | 55 | 5 | 30 | <0.0001 |
| **Pooled** |  |  |  |  |  |  |
| ***age-1(hx546)*** | **0 mM** | **120** | **74** | **46** | **22** | **N/A** |
| **data** | **50 mM** | **120** | **100** | **20** | **30** | **<0.0001** |
|  |  |  |  |  |  |  |
| *daf-2(e1370)* | 0 mM | 60 | 37 | 23 | 27 | N/A |
|  | 1 mM | 60 | 41 | 19 | 31 | 0.2763 |
|  | 10 mM | 62 | 46 | 16 | 27 | 0.9319 |
|  | 50 mM | 60 | 41 | 19 | 33 | 0.2907 |

**D. Lifespan data for *eat-2(ad1116)* treated with metformin**

|  | Metformin concentration | N total | N dead | N censored | Median lifespan (days) | *p* value  (log-rank) |
| --- | --- | --- | --- | --- | --- | --- |
| Trial 1 | 0 mM | 60 | 33 | 27 | 23 | N/A |
| (performed | 1 mM | 60 | 33 | 27 | 25 | 0.6589 |
| with wild-type | 10 mM | 60 | 33 | 27 | 21 | 0.5507 |
| Trial 1) | 50 mM | 60 | 32 | 28 | 23 | 0.8354 |
| Trial 2 | 0 mM | 60 | 34 | 26 | 22 | N/A |
| (performed | 1 mM | 60 | 38 | 22 | 20 | 0.247 |
| with wild-type | 10 mM | 60 | 38 | 22 | 18 | 0.011 |
| Trial 2) | 50 mM | 60 | 36 | 24 | 18 | 0.2182 |
| Trial 3 | 0 mM | 60 | 39 | 21 | 17 | N/A |
| (performed | 1 mM | 59 | 39 | 20 | 19 | 0.2013 |
| with wild-type | 10 mM | 60 | 39 | 21 | 19 | 0.5307 |
| Trial 3) | 50 mM | 61 | 39 | 22 | 17 | 0.6437 |
| Trial 4 | 0 mM | 60 | 30 | 30 | 23 | N/A |
| (performed | 1 mM | 59 | 32 | 27 | 25 | 0.7852 |
| with wild-type | 10 mM | 61 | 33 | 28 | 19 | 0.0050 |
| Trial 4) | 50 mM | 60 | 32 | 28 | 19 | 0.0033 |
| **Pooled** | **0 mM** | **240** | **136** | **104** | **21** | **N/A** |
| ***eat-2(ad1116)*** | **1 mM** | **238** | **142** | **96** | **21** | **0.9368** |
| **data** | **10 mM** | **241** | **143** | **98** | **19** | **0.0037** |
|  | **50 mM** | **241** | **139** | **102** | **19** | **0.0114** |

**E. Lifespan data for *aak-2* mutants treated with metformin**

|  | Metformin concentration | N total | N dead | N censored | Median lifespan (days) | *p* value  (log-rank) |
| --- | --- | --- | --- | --- | --- | --- |
| *aak-2(ok524)* | 0 mM | 60 | 50 | 10 | 17 | N/A |
| Trial 1 | 1 mM | 60 | 48 | 12 | 17 | 0.5101 |
| (Performed | 10 mM | 60 | 52 | 8 | 15 | 0.1862 |
| w/WT Trial 5) | 50 mM | 60 | 55 | 5 | 15 | 0.4589 |
| *aak-2(ok524)* | 0 mM | 59 | 52 | 7 | 19 | N/A |
| Trial 2 | 1 mM | 60 | 43 | 17 | 17 | 0.7492 |
| (Performed | 10 mM | 60 | 52 | 8 | 15 | 0.1839 |
| w/WT Trial 6) | 50 mM | 60 | 48 | 12 | 15 | 0.001 |
| *aak-2(ok524)* | 0 mM | 60 | 50 | 10 | 18 | N/A |
| Trial 3 | 1 mM | 60 | 47 | 13 | 16 | 0.6546 |
| (Performed | 10 mM | 60 | 46 | 14 | 16 | 0.1104 |
| w/WT Trial 7) | 50 mM | 60 | 49 | 11 | 16 | 0.0089 |
| **Pooled** | **0 mM** | **179** | **152** | **27** | **18** | **N/A** |
| ***aak-2(ok524)*** | **1 mM** | **180** | **138** | **42** | **17** | **0.5393** |
| **data** | **10 mM** | **180** | **150** | **30** | **16** | **0.0209** |
|  | **50 mM** | **180** | **152** | **28** | **16** | **<0.0001** |
| *aak-2(rr48)* | 0 mM | 60 | 46 | 14 | 13 | N/A |
| Trial 1 | 1 mM | 60 | 35 | 25 | 15 | 0.1311 |
|  | 10 mM | 60 | 45 | 15 | 15 | 0.0787 |
|  | 50 mM | 60 | 48 | 12 | 15 | 0.2711 |
| *aak-2(rr48)* | 0 mM | 60 | 49 | 11 | 17 | N/A |
| Trial 2 | 50 mM | 60 | 44 | 16 | 13 | 0.0002 |
| *aak-2(rr48)* |  |  |  |  |  |  |
| Trial 3 | 0 mM | 63 | 45 | 18 | 18 | N/A |
| (Performed | 50 mM | 60 | 52 | 8 | 16 | <0.0001 |
| w/WT Trial 10) |  |  |  |  |  |  |
| **Pooled** | **0 mM** | **183** | **140** | **43** | **16** | **N/A** |
| ***aak-2(rr48)*** | **1 mM** | **60** | **35** | **25** | **15** | **0.1239** |
| **data** | **10 mM** | **60** | **45** | **15** | **15** | **0.0442** |
|  | **50 mM** | **180** | **144** | **36** | **13** | **0.0004** |

**F. Lifespan data for *par-4* mutants treated** with metformin*

|  | Metformin concentration | N total | N dead | N censored | Median lifespan (days) | *p* value  (log-rank) |
| --- | --- | --- | --- | --- | --- | --- |
| *par-4(it47)* | 0 mM | 60 | 53 | 7 | 9 | N/A |
| Trial 1 | 50 mM | 60 | 53 | 7 | 9 | 0.0573 |
| *par-4(it47)* | 0 mM | 60 | 53 | 7 | 9 | N/A |
| Trial 2 | 50 mM | 60 | 51 | 9 | 9 | 0.0082 |
| *par-4(it47)* | 0 mM | 60 | 55 | 5 | 9 | N/A |
| Trial 3 | 50 mM | 60 | 54 | 6 | 9 | 0.4153 |
| **Pooled** |  |  |  |  |  |  |
| ***par-4(it47)*** | **0 mM** | **180** | **161** | **19** | **9** | **N/A** |
| **data** | **50 mM** | **180** | **158** | **22** | **9** | **0.0500** |
|  |  |  |  |  |  |  |
| *par-4(it57)* | 0 mM | 60 | 55 | 5 | 11 | N/A |
| Trial 1 | 50 mM | 61 | 59 | 2 | 11 | 0.4039 |
| *par-4(it57)* | 0 mM | 60 | 54 | 6 | 11 | N/A |
| Trial 2 | 50 mM | 60 | 46 | 14 | 9 | 0.0016 |
| *par-4(it57)* | 0 mM | 60 | 57 | 3 | 9 | N/A |
| Trial 3 | 50 mM | 60 | 51 | 9 | 9 | 0.8448 |
| *par-4(it57)* | 0 mM | 60 | 59 | 1 | 9 | N/A |
| Trial 4 | 50 mM | 60 | 60 | 0 | 9 | 0.8887 |
| **Pooled** |  |  |  |  |  |  |
| ***par-4(it57)*** | **0 mM** | **240** | **225** | **15** | **10** | **N/A** |
| **data** | **50 mM** | **241** | **216** | **25** | **9** | **0.0063** |
|  |  |  |  |  |  |  |
| Wild-type | 0 mM | 60 | 54 | 6 | 11 | N/A |
| (25°C) | 50 mM | 60 | 54 | 6 | 12 | 0.0038 |

*These studies conducted at 25°C, see Experimental Procedures

**G. Lifespan data for *skn-1(zu135)* treated with metformin**

|  | Metformin concentration | N total | N dead | N censored | Median lifespan (days) | *p* value  (log-rank) |
| --- | --- | --- | --- | --- | --- | --- |
| *skn-1(zu135)* | 0 mM | 60 | 46 | 14 | 9 | N/A |
| Trial 1 | 50 mM | 60 | 53 | 7 | 9 | 0.9837 |
| *skn-1(zu135)* | 0 mM | 60 | 53 | 7 | 11 | N/A |
| Trial 2 | 50 mM | 60 | 50 | 10 | 13 | 0.0595 |
| **Pooled** |  |  |  |  |  |  |
| ***skn-1(zu135)*** | **0 mM** | **120** | **99** | **21** | **11** | **N/A** |
| **data** | **50 mM** | **120** | **103** | **17** | **11** | **0.2733** |
| *skn-1(zu135)* |  |  |  |  |  |  |
| + *Is007[skn-* | 0 mM | 60 | 36 | 24 | 13 | N/A |
| *1::gfp]* | 50 mM | 60 | 46 | 14 | 20 | 0.0008 |
| Trial 1 |  |  |  |  |  |  |
| *skn-1(zu135)* |  |  |  |  |  |  |
| + *Is007[skn-* | 0 mM | 60 | 42 | 18 | 19 | N/A |
| *1::gfp]* | 50 mM | 60 | 46 | 14 | 21 | 0.0018 |
| Trial 2 |  |  |  |  |  |  |
| *skn-1(zu135)* |  |  |  |  |  |  |
| + *Is007[skn-* | 0 mM | 60 | 42 | 18 | 16 | N/A |
| *1::gfp]* | 50 mM | 60 | 37 | 23 | 16 | 0.0214 |
| Trial 3 |  |  |  |  |  |  |
| **Pooled** |  |  |  |  |  |  |
| ***skn-1(zu135)*** | **0 mM** | **180** | **120** | **60** | **16** | **N/A** |
| **+ *Is007[skn-*** | **50 mM** | **180** | **129** | **51** | **20** | **<0.0001** |
| ***1::gfp]*** |  |  |  |  |  |  |
| *skn-1(zu135)* |  |  |  |  |  |  |
| + *geIs9[gpa-* | 0 mM | 61 | 46 | 15 | 18 | N/A |
| *4p::skn-* | 50 mM | 60 | 34 | 26 | 13 | <0.0001 |
| *1b::gfp]* |  |  |  |  |  |  |
| Trial 1 |  |  |  |  |  |  |
| *skn-1(zu135)* |  |  |  |  |  |  |
| + *geIs9[gpa-* | 0 mM | 60 | 40 | 20 | 16 | N/A |
| *4p::skn-* | 50 mM | 60 | 50 | 10 | 16 | 0.0776 |
| *1b::gfp]* |  |  |  |  |  |  |
| Trial 2 |  |  |  |  |  |  |
| *skn-1(zu135)* |  |  |  |  |  |  |
| + *geIs9[gpa-* | 0 mM | 60 | 48 | 12 | 13 | N/A |
| *4p::skn-* | 50 mM | 60 | 50 | 10 | 13 | 0.0009 |
| *1b::gfp]* |  |  |  |  |  |  |
| Trial 3 |  |  |  |  |  |  |
| **Pooled** |  |  |  |  |  |  |
| ***skn-1(zu135)*** | **0 mM** | **181** | **134** | **47** | **16** | **N/A** |
| **+ *geIs9[gpa-*** | **50 mM** | **180** | **134** | **46** | **13** | **<0.0001** |
| ***4p::skn*** |  |  |  |  |  |  |
| ***-1b::gfp]*** |  |  |  |  |  |  |
| *skn-1(zu135)* |  |  |  |  |  |  |
| *+ geIs10[ges-* | 0 mM | 60 | 42 | 18 | 13 | N/A |
| *1p::skn-* | 50 mM | 61 | 43 | 18 | 15 | 0.1933 |
| *1c::gfp]* |  |  |  |  |  |  |
| Trial 1 |  |  |  |  |  |  |
| *skn-1(zu135)* |  |  |  |  |  |  |
| *+ geIs10[ges-* | 0 mM | 60 | 49 | 11 | 14 | N/A |
| *1p::skn-* | 50 mM | 60 | 49 | 11 | 16 | 0.0908 |
| *1c::gfp]* |  |  |  |  |  |  |
| Trial 2 |  |  |  |  |  |  |
| *skn-1(zu135)* |  |  |  |  |  |  |
| *+ geIs10[ges-* | 0 mM | 60 | 49 | 11 | 15 | N/A |
| *1p::skn-* | 50 mM | 60 | 44 | 16 | 13 | 0.5652 |
| *1c::gfp]* |  |  |  |  |  |  |
| Trial 3 |  |  |  |  |  |  |
| **Pooled** |  |  |  |  |  |  |
| ***skn-1(zu135)*** | **0 mM** | **180** | **140** | **40** | **13** | **N/A** |
| ***+geIs10[ges-*** | **50 mM** | **181** | **136** | **45** | **15** | **0.8764** |
| ***1p::skn-*** |  |  |  |  |  |  |
| ***1c::gfp]*** |  |  |  |  |  |  |
